# Autism-Associated PTCHD1 Missense Variants Bind to the SNARE-Associated Protein SNAPIN but Exhibit Impaired Subcellular Trafficking

**DOI:** 10.1016/j.bpsgos.2025.100492

**Published:** 2025-03-22

**Authors:** Stephen F. Pastore, Connie T.Y. Xie, Roya Derwish, Tahir Muhammad, Tereza Blahova, Sierra C. El-masri, Paul W. Frankland, Paul A. Hamel, John B. Vincent

**Affiliations:** a Molecular Neuropsychiatry & Development Lab, Molecular Brain Science Research Department, Campbell Family Mental Health Research Institute, Centre for Addiction and Mental Health, Toronto, Ontario, Canada; b Institute of Medical Science, University of Toronto, Toronto, Ontario, Canada; c Department of Laboratory Medicine & Pathobiology, University of Toronto, Toronto, Ontario, Canada; d Program in Neurosciences and Mental Health, The Hospital for Sick Children, Toronto, Ontario, Canada; e Department of Physiology, University of Toronto, Toronto, Ontario, Canada; f Department of Psychology, University of Toronto, Toronto, Ontario, Canada; g Department of Psychiatry, University of Toronto, Toronto, Ontario, Canada

**Keywords:** Autism spectrum disorder, Clinical missense variant, Intellectual disability, Protein-protein interaction, SNARE complex, Transmembrane protein trafficking

## Abstract

**Background:**

*PTCHD1* is a susceptibility gene for autism spectrum disorder and intellectual disability. Its function in brain development and neurotransmission remains elusive. Studies have sought to characterize PTCHD1 function by elucidating its neural network of interacting proteins. However, given the current paucity of functional information, many PTCHD1 missense variants in clinical databases are classified as variants of uncertain significance (VUSs), severely limiting the health care resources available to patients and families.

**Methods:**

A yeast 2-hybrid assay was used to identify synaptic PTCHD1-interacting proteins. Candidate binding partners were validated by cloning; transient overexpression in human embryonic kidney (HEK) 293T cells, followed by co-immunoprecipitation and immunoblotting; and immunocytochemistry in differentiated P19 cells. Site-directed mutagenesis was used to evaluate the pathogenicity of clinical missense variants, followed by transient overexpression and immunocytochemistry in non-neuronal (HEK293T) and neuronal (Neuro-2a cells) systems.

**Results:**

A novel interaction was identified between the first lumenal loop of PTCHD1 and the SNARE-associated protein SNAPIN, which is implicated in synaptic vesicle exocytosis. Clinically associated missense variants within this region did not disrupt SNAPIN binding, indicating that the pathoetiology of these variants is unrelated to this interaction. However, 6 of the 12 missense variants tested exhibited pronounced retention within the endoplasmic reticulum and impaired neuronal and non-neuronal trafficking to the plasma membrane.

**Conclusions:**

These data yield insights into the role of PTCHD1 in neurodevelopment and neurotransmission and suggest a neuropathological mechanism for missense variants. These findings provide a platform for diagnostic assay and VUS interpretation, allowing for clinical reclassification of these variants.

## Plain Language Summary

Loss-of-function variants in the X-linked *PTCHD1* gene result in autism spectrum disorder and/or intellectual disability in males. However, missense variants in *PTCHD1* are typically reported as variants of uncertain significance by diagnostic laboratories, in part due to the limited understanding of PTCHD1 protein function and the lack of a functional assay. Pastore *et al.* (article#) identify the SNARE-associated protein SNAPIN as a candidate protein interacting partner for PTCHD1 through PTCHD1 external loop 1. However, clinically reported missense variants in loop 1 do not impact the protein-protein interaction. Of 12 PTCHD1 missense variants tested, 6 exhibited pronounced retention within the endoplasmic reticulum and impaired neuronal and non-neuronal trafficking to the plasma membrane, suggesting a putative (and assayable) neuropathological mechanism.

Autism spectrum disorder (ASD) is a heterogeneous neurodevelopmental disorder typified by social and communicative deficiencies, restricted interests, and repetitive sensorimotor behaviors (1). ASD exhibits 50% to 80% comorbidity with intellectual disability (ID) (2). Genetics appear to strongly influence the pathoetiology of ASD, with a recent meta-analysis concluding that 74% to 93% of ASD risk is heritable (3). Genome-wide studies of affected cohorts have identified numerous ASD and ID susceptibility genes. *PTCHD1*, on Xp22.11, was first implicated in the pathoetiology of ASD and ID in 2008 (4). Numerous highly penetrant rare genomic deletion variants and loss-of-function coding variants have subsequently been detected in *PTCHD1* (5), and many rare missense variants have been identified through clinical diagnostics (for example, see ClinVar: http://www.ncbi.nlm.nih.gov/clinvar).

In silico analyses have predicted PTCHD1 to be a multipass transmembrane protein consisting of 12 transmembrane domains (TMDs). Exogenous PTCHD1 has been found to localize within cell membranes (6, 7, 8) and to localize to the postsynaptic density (PSD) dendritic spines of neurons (7). In addition, PTCHD1 is predicted to possess 2 distinct sterol sensing domain-like modules, 2 large lumenal loops, and a C-terminal PDZ-binding domain (5). Functionally, a definitive cellular role for PTCHD1 has yet to be elucidated. PTCHD1 exhibits 21.17% sequence homology with the cholesterol-trafficking transmembrane protein Niemann-Pick disease, type C1 (5), and has correspondingly been reported to directly bind cholesterol (9). To delineate a putative function of PTCHD1, previous research has sought to characterize its network of interacting proteins in neurons. These studies have used affinity purification to identify and validate several putative binding partners of PTCHD1 in vitro. In this regard, both PSD95 and SAP102 have been reported to interact with PTCHD1 via its C-terminal PDZ-binding domain (7,10).

Although estimating the proportion of ASD and ID attributable to PTCHD1 variants is difficult, using the Autism Speaks/MSSNG dataset (accessed December 2024) and using only good-quality hemizygous calls in males with ASD, we estimated that a frequency of 0.32% have a PTCHD1 variant (*N* = 4762, including affected siblings; loss copy number variants [CNVs] spanning 1 or more PTCHD1 exons [but not multigenic]: *n* = 3; rare hemizygous missense: *n* = 12 [6 of which have supportive functional evidence in this study and others]). For the DECIPHER dataset (which includes all genetic/genomic developmental disorders [DDs], both male [55%] and female [45%], *N* = 36,000 [*n* ∼ 19,800 male]) (https://www.deciphergenomics.org/about/overview), hemizygous variants at PTCHD1 account for 0.1% of all male DD cases (*n* = 12 loss CNVs, *n* = 4 pathogenic loss of function and *n* = 4 missense variants of uncertain significance [VUSs]).

More than 300 rare missense variants have been identified in *PTCHD1* through clinical diagnostics in autistic individuals and individuals with ID (11,12). However, molecular mechanisms of pathology exist for a very small subset of these variants (8,13,14), and the vast majority are interpreted as VUSs due to the lack of supporting segregation or functional data. This diagnostic uncertainty negatively impacts the integrative health care options available to many families. Functional evidence from molecular studies that would enable reclassification of PTCHD1 variants from VUSs to “likely pathogenic” would provide an explanation for individual challenges and enable thousands of families to access additional health care resources, including testing of carrier status in female relatives and appropriate genetic counseling.

The objective of the current study was to agnostically identify additional synaptic proteins that can interact with PTCHD1, beyond interactions through its C-terminal PDZ-binding domain, in order to elaborate on its potential function(s) in neurons. Furthermore, we sought to evaluate PTCHD1 VUSs in both neuronal and non-neuronal systems in order to provide evidence for their possible clinical reclassification.

## Methods and Materials

A full description of the methods is given in the Supplement.

### Yeast 2-Hybrid Screen

The Matchmaker Gold Yeast Two-Hybrid System (Takara Bio) was used. Briefly, lumenal loop 1 (amino acids p.Glu49-p.Arg270) or a fusion of loop 1 to lumenal loop 2 (amino acids p.Gln521-p.Ser695) of human *PTCHD1* was cloned into pGBKT7 in-frame with the DNA-binding domain of transcription factor Gal4. Constructs were separately transformed into the *S. cerevisiae* strain Y2HGold and independently used as bait to probe 2 separate complementary DNA (cDNA) libraries fused to the activation domain of Gal4: 1) Mate & Plate Library Mouse Embryo Day 11 and 2) Normalized Mate & Plate Library Adult Human Brain (Takara Bio). Three independent screens were performed for each library. Positive clones were selected on synthetic dropout medium in the absence of tryptophan, leucine, histidine, and adenine and supplemented with X-gal. For positive clones, plasmid DNA was extracted and transformed into *E. coli*, and then individual colonies were amplified by polymerase chain reaction (PCR) and Sanger sequenced to identify the insert.

### Cloning

The complete coding sequences of mouse *Ptchd1* and *Snapin*, which exhibit 98.1% and 97.8% protein sequence homology with their human orthologs, respectively, were cloned into pcDNA3.1-myc-HisB. For *Ptchd1*, the stop codon was included, and 3xFlag was subsequently inserted N-terminally. Finally, 3xFlag-tagged *Ptchd1* lumenal loop 1 (p.Val48-p.Arg266), lumenal loop 2 (p.Tyr499-p.Ala698), and a loop 1-loop 2 chimeric protein were cloned into pcDNA3.1-myc-HisB, succeeded by a stop codon. Human *PTCHD1* was recombined into pDEST53 in-frame with GFP (green fluorescent protein), and human SNAPIN cloned into pcDNA3.1-myc-HisB. Primer sequences are provided in Table S3.

Site-directed mutagenesis was used to generate *Ptchd1* missense variants (Table 1). The amino acid residues for all 14 missense variants are conserved between human and mouse (see Figure S5). Briefly, wild-type (WT) *3xFlag-Ptchd1-pcDNA3.1* was amplified using Q5-high-fidelity DNA polymerase, with forward primer containing the mutant codon. Next, purified PCR products were phosphorylated and recircularized with DNA ligase. All mutant constructs were confirmed by Sanger sequencing (Figures S1 and S2). Primer sequences are shown in Table S4.

**Table 1:** Location and Clinical Information for PTCHD1 Missense Variants

Variant	Location	Clinical Features	Source
P32R	TMD 1	ASD, ID, congenital ataxia	Halewa *et al.*, 2021 (8)
S51N	Loop 1	ID	Torrico *et al.*, 2015 (18)
73F		ASD	Noor *et al.*, 2010 (6)
P75L		ASD	MSSNG-AU3951302
P75Q		ASD	MSSNG-AU3794302
Q102R		ID	Personal communication
V150M		Susceptibility to ASD	ClinVar-1334078
K181T		ASD, epilepsy	Karaca *et al.*, 2015 (19)
V195I		ASD, severe language delay	Noor *et al.*, 2010 (6)
Y213C		Developmental delay	Halewa *et al.*, 2021 (8)
G303R	TMD 3 and SSD	Susceptibility to ASD	ClinVar-417957
D527E	Loop 2	ASD	Firth *et al.*, 2009 (20)
F549C		ASD	Personal communication
T602A		Unknown	ClinVar-1303783

Five loop-1 variants were selected, 3 loop-2 variants, plus 1 variant in TMD1 and 1 in TMD3 that is also within the SSD. For MSSNG data, see https://research.mss.ng; for ClinVar data, see https://www.ncbi.nlm.nih.gov/clinvar.

ASD, autism spectrum disorder; ID, intellectual disability; SSD, sterol sensing domain; TMD, transmembrane domain.

### Cell Culture and Neuronal Differentiation

Human embryonic kidney (HEK) 293T cells were maintained in Dulbecco’s Modified Eagle Medium (DMEM) with 10% fetal bovine serum (FBS) and 1% penicillin-streptomycin (PS). Cells were passaged every 48 to 72 hours at semiconfluence by trypsinization. P19 mouse embryonal carcinoma cells were maintained in α-MEM with 7.5% newborn calf serum, 2.5% FBS, and 1% PS and passaged every 48 to 72 hours at semiconfluence. For immunocytochemical staining, Lipofectamine 3000 was utilized to cotransfect *GFP-Ptchd1* and *Snapin-myc* into the cells. To induce neuronal differentiation, cells were grown on plates with 1 μM all-trans retinoic acid (RA). On the fourth day, aggregates were plated onto coverslips to differentiate for 6 days. Mouse Neuro-2a (N2a) neuroblastoma cells were seeded on coverslips and then cultured in DMEM with high glucose and 10% FBS with 1% antibiotics. For transfections, DNA constructs (WT or mutant *3xFlag-Ptchd1* plus *Snapin-myc* [see above]) were mixed with 0.1% polyethyleneimine (15) and added to N2a cells. Subsequently, neuronal differentiation was induced by switching media to 4% FBS and adding RA (final concentration 2 μM).

### Co-Immunoprecipitation

Reseeded HEK293T cells were cotransfected using Lipofectamine 3000 with 2 μg of *3xFlag-Ptchd1* and 500 ng of *Snapin-myc* expression plasmids. After incubation/washing, cells were lysed in immunoprecipitation (IP) buffer, incubated, and then centrifuged. Supernatants were quantified by Bradford assay; 500 μg of lysates were incubated with 1 μg of mouse α-Flag M2 antibody in IP buffer and then incubated with Dynabeads ProteinG (Thermo Fisher Scientific). Antibody-protein complexes were separated magnetically, washed, and then eluted. Cotransfected lysates were immunoprecipitated using mouse anti-Flag antibody, and subsequent immunoblots were probed for Flag using rabbit anti-Flag antibody (Table S5). Two biological replicates were performed for co-IP experiments.

### Immunoblotting

Input lysates and elution products were combined with 4× Laemmli buffer with 10% β-mercaptoethanol and then incubated at 37 °C (20 minutes) to avoid boiling-induced aggregation (16). Samples were separated using 4% to 15% gradient sodium dodecyl sulfate polyacrylamide gel electrophoresis gels, transferred to polyvinylidene difluoride membranes, and blocked (3% skimmed milk powder). Blots were incubated with primary antibodies, blocked, washed, and then incubated with horseradish peroxidase–conjugated secondaries in blocking solution and washed again, followed by chemiluminescence detection.

### Immunocytochemistry

HEK293T cells on coated coverslips were cotransfected with 10 ng of *3xFlag-Ptchd1* and 490 ng of inert carrier plasmid pBV-Luc, using Lipofectamine 3000. After rinse/wash/blocking steps, cells were incubated with primary antibodies. After washing, cells were incubated with AlexaFluor-conjugated secondary antibodies. After washing/incubation, cells were mounted on glass slides using Dako mounting medium. Three biological replicates were performed. For transfected N2a cells, cells grown on coverslips were fixed, permeabilized, blocked, and then incubated with primary antibodies. After washing steps, cells were incubated with AlexaFluor-conjugated secondaries, washed, and mounted onto slides using VectaShield Mounting media/DAPI, and images were taken using the Nikon Eclipse 80*i* fluorescence microscope using QICAM-UV Fast 1394 and QCapture Suite PLUS software.

### Quantification of Colocalization Ptchd1 in HEK293 Cells

Images were acquired using a Leica TCS SP8 confocal microscope with Leica Application Suite X software. The 405 nm (66.6% intensity), 488 nm (5.2% intensity), and 552 nm (2.0% intensity) lasers were used. Images were acquired sequentially under 63× magnification. For each image, consecutive high-resolution z-stacks were acquired with a z-interval of 0.6 μm. For each variant, 3 biological replicates (each consisting of 3 consecutive z-stacks for 6 to 7 separate images) were blindly analyzed by an independent technician. Pixel intensity thresholds were adjusted identically for all images: 1) 3xFlag-Ptchd1 (minimum 30, maximum 255) and 2) endoplasmic reticulum (ER) marker calnexin (CNX) or cell membrane marker ATP1A1 (minimum 15, maximum 230). To quantify the overlapping intensity values of 3xFlag-Ptchd1 and CNX or ATP1A1, Pearson correlation coefficients were computed with the JACoP (17) plugin in FIJI using default settings. To calculate statistical significance, a 1-way analysis of variance was used, followed by Tukey’s honestly significant difference test to compare variants of Ptchd1 with WT.

## Results

### Identification of SNAPIN as a PTCHD1-Binding Protein

**Figure 1:**
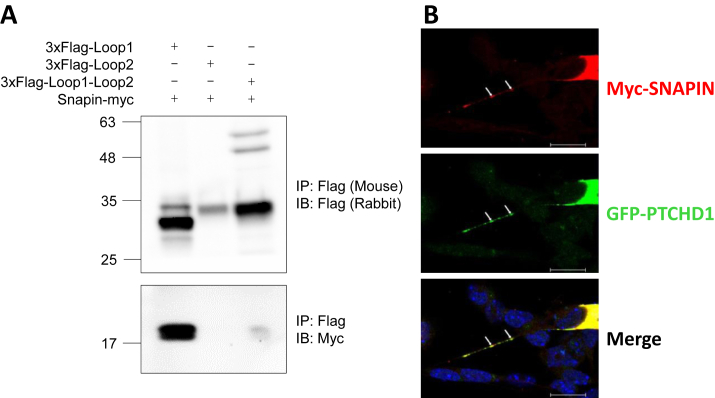
Protein interaction and neuronal colocalization between PTCHD1 and SNAPIN. **(A)** Immunoblot image following co-IP of 3xFlag-tagged mouse Ptchd1 lumenal loop 1, lumenal loop 2, or a lumenal loop 1-loop 2 chimera (top blot); and myc-tagged mouse Snapin (bottom blot). **(B)** Immunocytochemical staining of transiently expressed GFP-tagged human PTCHD1 and myc-tagged human SNAPIN in P19-induced neurons after 6 days of differentiation. Arrows indicate a region along the dendritic process where PTCHD1 and SNAPIN exhibit colocalization. Scale bar = 20 μm. GFP, green fluorescent protein; IB, immunoblotting; IP, immunoprecipitation.

To identify novel neural proteins that may putatively interact with predicted exoplasmic domains of PTCHD1, lumenal loop 1 or a fusion of lumenal loops 1 and 2 were separately used as bait in yeast 2-hybrid screens. Subsequent sequencing of positive clones from the screen of loop 1 against an adult human brain cDNA library indicated a highly successful bait-prey interaction between this region of PTCHD1 and SNAPIN, with 17 hits, compared with the next-highest candidate protein, with 2 hits (Table S1). This result was confirmed via co-IP, which revealed that mouse Snapin interacted strongly with the mouse Ptchd1 loop 1 and very weakly with the loop 1-loop 2 fusion protein (Figure 1A). To exclude the possibility that the binding between human PTCHD1 and human SNAPIN is an artificial byproduct of their spontaneous proximity to one another during cell lysis or ectopic expression in yeast cells, immunocytochemistry was used to evaluate their subcellular localization in P19-induced neural cells. Both PTCHD1 and SNAPIN were observed to colocalize within dendritic projections (Figure 1B).

### Binding of Snapin to Clinical Variants

**Figure 2:**
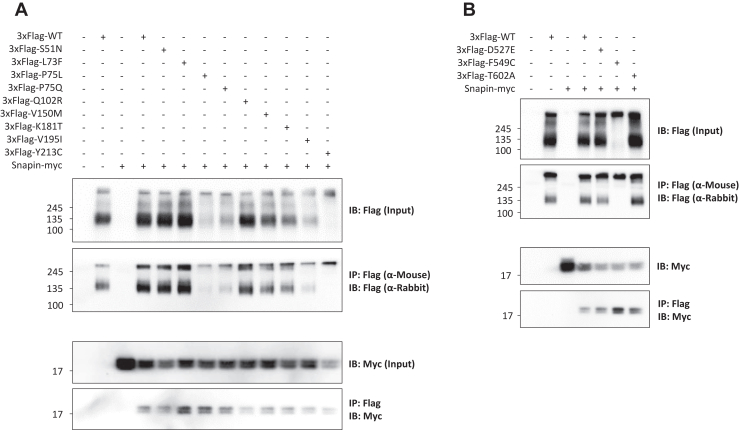
Protein interaction of clinical lumenal loop missense variants and Snapin immunoblot images following co-IP of 3xFlag-tagged Ptchd1 **(A)** lumenal loop 1 and **(B)** lumenal loop 2 missense variants and myc-tagged Snapin. Input blots are shown above their respective Flag-immunoprecipitated blots. IB, immunoblotting; IP, immunoprecipitation; WT, wild-type.

*PTCHD1* single nucleotide variants evaluated in this study were identified in the literature (6,8,18,19), in clinical genomics databases (11,20), and through personal communication with clinicians (Table 1). Two of these variants, p.Gln102Arg and p.Val150Met, were identified in multiplex ID Pakistani families (Figures S3 and S4, respectively). To ascertain whether the mechanism of pathogenicity for clinically identified point mutations within *PTCHD1* is related to an impaired capacity to bind SNAPIN, co-IP experiments were performed with these variants using tagged mouse *Ptchd1* and *Snapin* expression constructs in HEK293T cells. It was observed that all lumenal loop variants evaluated in this study were both detectable at the protein level and able to interact with Snapin (Figure 2). Variations in monomeric protein expression were observed, with the variants p.Pro75Leu, p.Pro75Gln, p.Val195Ile, p.Tyr213Cys, and p.Phe549Cys exhibiting attenuated protein levels at the expected size relative to WT Ptchd1 (Figure 2B). The 2 TMD variants, p.Pro32Arg and p.Gly303Arg, were not tested experimentally here, because previous studies had shown reduced expression levels for constructs with these mutations (10,16).

### Neuronal and Non-neuronal Subcellular Localization of Clinical Variants

**Figure 3:**
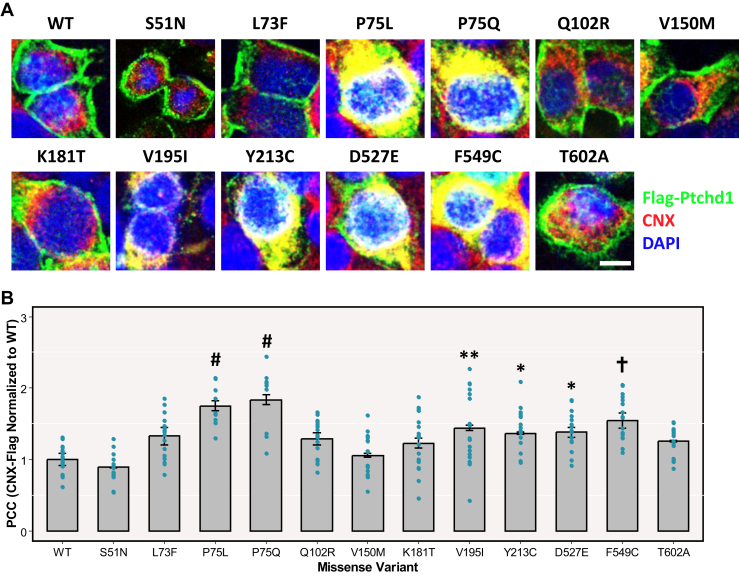
ER retention of clinical missense variants. **(A)** Immunocytochemical staining of exogeneous Flag-Ptchd1 (green), endogenous CNX (ER marker; red signal), and DAPI (nuclear marker; blue signal) in HEK293T cells. Representative merged fluorescent images from 3 independent experiments were taken for WT and each missense variant. **(B)** PCCs, calculated by JACoP, of overlapping pixel intensities between transiently expressed 3xFlag-tagged Ptchd1 and endogenous CNX (ER marker). Data are expressed as the mean ± SEM, and each missense variant was normalized to WT Ptchd1. Individual data points for all technical replicates are plotted. Data were analyzed using 1-way analysis of variance followed by Tukey’s honestly significant difference test (∗*p* < .05, ∗∗*p* < .01, ^✝^*p* < .001, ^#^*p* < .0001; *n* = 3 immunocytochemical costaining experiments from 3 separate transfections with 6 to 7 images captured per staining; *df* = 2). Scale bar = 10 μm. CNX, calnexin; ER, endoplasmic reticulum; HEK, human embryonic kidney; PCC, Pearson correlation coefficient; WT, wild-type.

**Figure 4:**
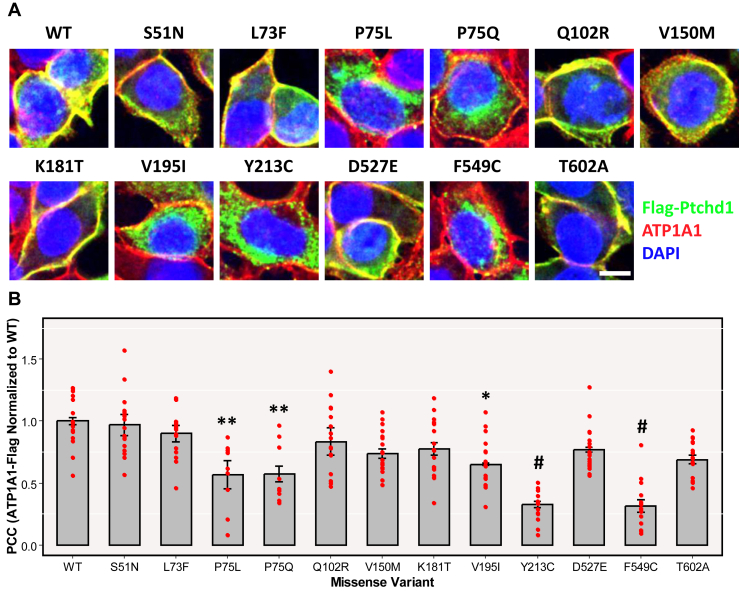
Membrane trafficking of clinical missense variants. **(A)** Immunocytochemical staining of exogeneous Flag-Ptchd1 (green signal), endogenous ATP1A1 (membrane marker; red signal), and DAPI (blue signal) in HEK293T cells. Representative merged fluorescent images from 3 independent experiments were taken for WT and each missense variant. **(B)** PCCs, calculated by JACoP, of overlapping pixel intensities between transiently expressed 3xFlag-tagged Ptchd1 and endogenous ATP1A1 (membrane marker) in HEK293T cells. Data are expressed as the mean ± SEM, and each missense variant was normalized to WT Ptchd1. Individual data points for all technical replicates are plotted. Data were analyzed using 1-way analysis of variance followed by Tukey’s honestly significant difference test (∗*p* < .05, ∗∗*p* < .01, ^#^*p* < .0001; *n* = 3 immunocytochemical costaining experiments from 3 separate transfections with 6 to 7 images captured per staining; *df* = 2). Scale bar = 10 μm. HEK, human embryonic kidney; PCC, Pearson correlation coefficient; WT, wild-type.

Because the PTCHD1 VUS missense variants do not appear to hinder binding of PTCHD1 to SNAPIN, our efforts to understand their relationship to pathogenicity were focused next on evaluation of their subcellular localization. Upon transient transfection in HEK293T cells, 6 of the 12 lumenal point mutants, p.Pro75Leu, p.Pro75Gln, p.Val195Ile, p.Tyr213Cys, p.Asp527Glu, and p.Phe549Cys, were all observed to demonstrate increased retention within the ER compared with WT (Figure 3A). Mutations at p.Pro75 appear to be particularly deleterious, with p.Pro75Leu and p.Pro75Gln exhibiting 75% and 84% higher associations with the ER marker CNX, respectively, than WT (Figure 3B). Correspondingly, all of these variants, with the exception of p.Asp527Glu, were associated with attenuated localization within the cell membrane relative to WT (Figure 4A). Variants generating cysteine residues, which are undesirable because of the potential to generate additional disulfide bridging and thus impact 3-dimensional (3D) structure, seemingly impaired cell membrane trafficking to a significant extent, with p.Tyr213Cys and p.Phe549Cys displaying 67% and 68% less colocalization with the cell membrane marker ATP1A1, respectively, than WT (Figure 4B). Similarly, mutations affecting p.Pro75 (proline residues are particularly rigid within a 3D structure) considerably impaired the ability of these point mutants to be trafficked to the cell membrane because colocalization with ATP1A1 was attenuated in p.Pro75Leu and p.Pro75Gln by 43% and 42%, respectively, relative to WT (Figure 4B).

**Figure 5:**
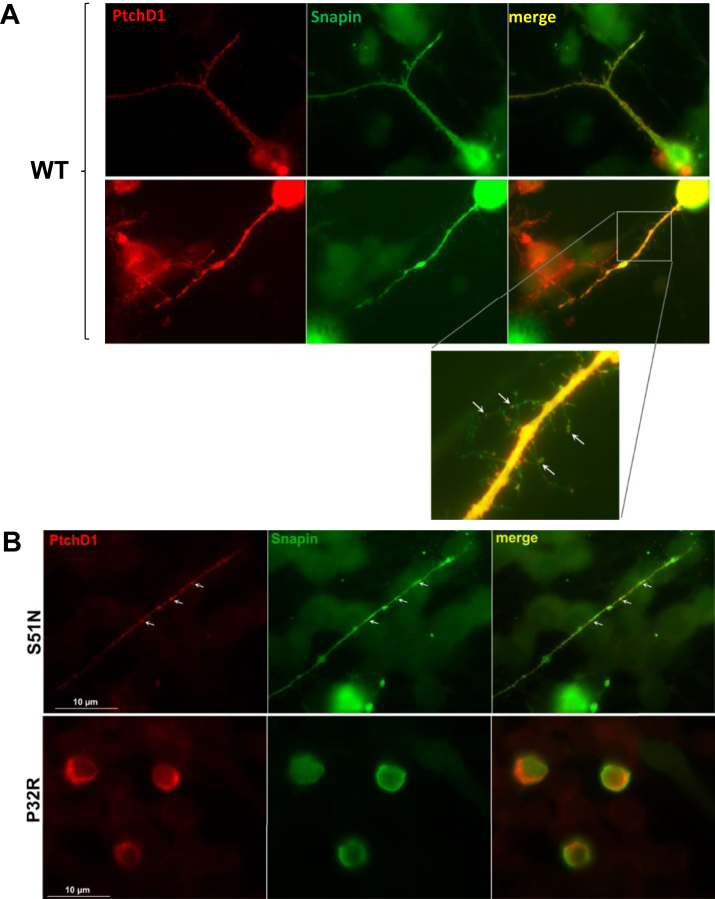
Localization of Ptchd1 and Snapin in N2a cells. WT Ptchd1 **(A)** or Ptchd1 missense variants **(B)** were coexpressed with Snapin in N2a cells. **(A)** WT Ptchd1 localizes to neuronal processes appearing in a punctate pattern along processes. The enlarged inset shows Ptchd1 localizes in discrete structures in neuronal spikes along the length of these processes. Snapin also localizes to the same structures but in a diffuse pattern that, while overlapping, does not appear as specific puncta. **(B)** Variants of Ptchd1 have distinct localization qualities. A subset of variants, such as S51N (upper panels), localize in a manner that is indistinguishable from WT Ptchd1. A distinct subset of Ptchd1 variants, including P32R (lower panels), were found localized only to the cell body of N2a cells and not in neuronal processes (see Table S5 for a summary of all missense variant data). N2a, Neuro-2a; WT, wild-type.

Next, we sought to characterize trafficking of lumenal loop 1 and 2 variants (plus p.Pro32Arg and p.Gly303Arg from TMD1 and TMD3, respectively) in a neuronal system. Transfections of these variants into N2a cells revealed categorical patterns of localization (Figure 5). Transient expression of WT Ptchd1 results in localization to neuronal processes of N2a cells. In these processes, Ptchd1 appears in a punctate pattern along the processes as well as in the small spikes that emanate perpendicular to these processes (Figure 5A). While Snapin expression is also observed in these same processes, Snapin is confined to the middle of the processes, and specific colocalization with punctate pattern of WT Ptchd1 is not evident. The localization of various Ptchd1 variants was then assessed. Two specific patterns of expression were observed for the variants. Representative images of these cellular expression patterns, with p.Ser51Asn and p.Pro32Arg as the examples, are shown in Figure 5. The variants p.Ser51Asn (Figure 5B, upper panels), p.Gln102Arg, p.Val195Ile, and p.Asp527Glu trafficked to plasma membranes of both the cell body and dendrites in a punctate pattern indistinguishable from WT Ptchd1. A distinct category of localization was seen for other variants, as illustrated by the p.Pro32Arg variant (Figure 5B, lower panels). These variants were confined to the cell body and showed no evidence of trafficking to the processes when transiently expressed. Variants that behaved in this manner included the lumenal variants p.Pro75Gln, p.Val150Met, p.Tyr213Cys, and p.Phe549Cys, as well as the transmembrane variants (Table 2).

**Table 2:** Allele Frequencies and Clinical Predictions of PTCHD1 Missense Variants and Summary of Subcellular Trafficking of Missense Variants in Neuronal Cells

DNA Variant	SNP ID	Protein Consequence	Location in PTCHD1	Clinical Significance	Allele Frequency (No. of Hemizygotes)	Membrane Localization	Dendrite Localization
WT	–	–	–	–	–	+	Punctate pattern
23334970 C > G	–	p.Pro32Arg	TMD1	Likely pathogenic	8.27 × 10^−7^ (0)	−	−
23335027 G > A	–	p.Ser51Asn	Loop 1	VUS	9.11 × 10^−7^ (0)	+	Punctate pattern
23335092 C > T	rs373105249	p.Leu73Phe	Loop 1	Conflicting classifications	6.65 × 10^−5^ (21)		
23335099 C > A	–	p.Pro75Gln	Loop 1	VUS	0 (0)	−	+
23335099 C > T	rs1198691680	p.Pro75Leu	Loop 1	VUS	8.94 × 10^−6^ (1)		
23335180 A > G	rs138264321	p.Gln102Arg	Loop 1	VUS	7.29 × 10^−6^ (4)	+	Punctate pattern
23379687 G > A	rs747642714	p.Val150Met	Loop 1	VUS	7.44 × 10^−6^ (3)	−	−
23379781 A > C	rs1175938564	p.Lys181Thr	Loop 1	Likely pathogenic	9.11 × 10^−7^ (1)		
23379822 G > A	rs769407241	p.Val195Ile	Loop 1	VUS	5.65 × 10^−5^ (25)	+	Punctate pattern
23379877 A > G	–	p.Tyr213Cys	Loop 1	Conflicting classifications	0 (0)	−	+
23380146 G > A	rs1060499615	p.Gly303Arg	TMD3	VUS	0 (0)	−	−
23393099 C > A	–	p.Asp527Glu	Loop 2	VUS	1.91 × 10^−5^ (11)	+	Punctate pattern
23393164 T > G	–	p.Phe549Cys	Loop 2	VUS	0 (0)	−	−
23393322 A > G	–	p.Thr602Ala	Loop 2	VUS	1.82 × 10^−6^ (0)		

Here, we have summarized the subcellular trafficking results for loop 1 and loop 2 missense variants in differentiated neuronal cells (Neuro-2a); for comparison, TMD missense variants p.Pro32Arg and p.Gly303Arg were also used. Statistical genetic and clinical information for missense variants assayed in this study; genomic coordinates corresponding to the GRCh38 assembly; and predictions of clinical significance are taken from ClinVar. Allele frequencies and numbers of hemizygotes were obtained from gnomAD version 4.1.0 (34).

SNP, single nucleotide polymorphism; TMD, transmembrane domain; VUS, variant of uncertain significance; WT, wild-type.

Taken together, while we have shown the association of Ptchd1 with the important adapter of SNARE complexes Snapin, missense variants of Ptchd1 derived from patients with ASD/ID exhibit distinct patterns of subcellular localization. In the case of neurons, a number of these variants fail to localize to the neuronal processes where it is expected that Ptchd1 activity is important, thus effectively causing a functional knockout of Ptchd1 in these cells.

## Discussion

Due to the paucity of definitive functional information for PTCHD1, studies have sought to deduce its role by delineating its network of interacting proteins. Affinity purification experiments with adult mouse neural lysates indicate that Ptchd1 binds with the PSD proteins Sap102, Psd95, Dlg1-3, Magi1, Magi3, and Lin7, as well as with components of the retromer complex Snx27, Vps35, and Vps26b (7,10). Of these, the only interactions with Ptchd1 that have been reported to occur independent of the C-terminal PDZ-binding domain is with the retromer complex proteins Vps35 and Vps26b (10). Given the predicted multipass transmembrane structure and membrane topology of PTCHD1, it is plausible that in addition to the cytoplasmic C-terminus, the 2 large lumenal loops may facilitate further protein-protein interactions. In this regard, the current study has identified a novel binding partner, SNAPIN, which demonstrates a high-affinity interaction with the first lumenal loop of PTCHD1.

SNAPIN associates with SNAP25 (21), which is a core component of the *trans*-SNARE complex, together with synaptobrevin and syntaxin (22). The *trans*-SNARE complex facilitates the fusion of exocytic vesicles, as well as the exocytosis of neurotransmitter-containing synaptic vesicles following depolarization of presynaptic neurons (23). In *C. elegans*, binding of the SNAPIN ortholog SNPN-1 to SNAP25 stabilizes SNARE complex formation and promotes synaptic vesicle priming (24). Functionally, primary cortical neurons from Snapin^null^ mice show reduced frequency of miniature excitatory postsynaptic currents, a smaller pool of release-ready synaptic vesicles, and desynchronized synaptic vesicle fusion (25). In addition, at the neuromuscular junctions of snpn-1^null^ *C. elegans*, the amount of docked, fusion-competent vesicles was significantly reduced, although the overall kinetics of synaptic transmission were unaffected (24). Finally, the dosage of Snapin in mouse primary hippocampal neurons appears to influence dendritic arborization by its binding to the cytosolic PSD95 interactor Cypin (mouse ortholog of human guanine deaminase) (26). Collectively, these data indicate that SNAPIN is intimately involved in both neurodevelopment and neurotransmission. Developmentally, Snapin^null^ mutant mice exhibit embryonic lethality (24), whereas Ptchd1 mutant mice are viable, but demonstrate an array of neurodevelopmental abnormalities and ASD-like behaviors (5). This disparity indicates that in the absence of Ptchd1, Snapin still maintains a basal level of activity sufficient to ensure embryonic survival. Studies have indicated that dysregulation of neurotransmission is implicated in the pathoetiology of ASD (27). Therefore, PTCHD1 may be involved in coordinating SNAPIN-mediated synaptic vesicle exocytosis, contributing to the precise synchrony of neurotransmission within the brain that is required for proper cognition.

SNAPIN also has well-established cytoplasmic functions. For example, it is known to bind to dynein—cytoskeletal motors involved in intracellular trafficking—and the Snapin-dynein intermediate chain (DIC) interaction is critical for retrograde transport of TrkB (tropomyosin receptor kinase B)–signaling endosomes from axon terminals to the neuronal cell body. Disruption of this interaction impairs Bdnf signaling and reduces growth of cortical dendrites (28). Although we do not yet know whether the Snapin-DIC interaction is also involved in retrograde transport of Ptchd1, this may also represent an opportunity for Ptchd1-Snapin interaction.

Identification of SNAPIN as a putative PTCHD1 binding partner was obtained from a yeast 2-hybrid screen. An inherent limitation of this in vitro approach is that it provides artificial opportunities for protein interaction, potentially resulting in false positives because the candidate proteins are expressed ectopically within the same subcellular compartment. Snapin has been found to be expressed ubiquitously in various rat brain subregions, including both the cerebellum and cortex (21), where *PTCHD1* is abundant at the transcript level (5). Furthermore, in fractionated rat cerebral synaptosomes, Snapin was exclusively detected in synaptic vesicles (21). Together, these data are consistent with the subcellular colocalization that we observed between PTCHD1 and SNAPIN within the dendritic processes of P19-induced neurons in this study (Figure 1B). In addition to this neuronal colocalization, the validated binding between Snapin and the first lumenal loop of Ptchd1 (Figure 1A) suggests the likelihood of a genuine functional interaction.

This study focused primarily on nonsynonymous variants within the soluble lumenal loops of PTCHD1. Ten of the lumenal missense variants were detectable in monomeric form at varying levels (Figure 2). This is in contrast to Halewa *et al.* (8), where marked instability of the p.Lys181Thr point mutant was reported, a result that was not observed in this study (Figure 2). This discrepancy may be attributable to differences in the epitope tag, transfection conditions, or the length of the posttransfection incubation period prior to cell lysis. Furthermore, the 2 missense variants that generated noncanonical cysteine residues, p.Tyr213Cys and p.Phe549Cys, were only detectable in aggregate form (Figure 2), possibly due to the formation of disulfide bridge cross-linked homodimers. Finally, the 2 variants involving mutated proline residues, p.Pro75Leu and p.Pro75Gln, as well as the variant p.Val195Ile, appeared to demonstrate reduced monomeric protein expression (Figure 2). Within a protein, the conformational rigidity of proline residues confers unique local secondary structure, with the cyclic nature of proline side chains anchoring the dihedral angle ϕ at approximately −65° (29). Consequently, substitutions that generate or abolish proline residues may result in relatively pronounced local conformational changes compared to other types of substitutions (30), leading to protein misfolding and aggregation.

Immunocytochemical analyses revealed that 6 missense variants (p.Pro75Leu, p.Pro75Gln, p.Val195Ile, p.Tyr213Cys, p.Asp527Glu, and p.Phe549Cys) showed apparent retention by the ER (Figure 3). Consistent with this, 5 of these ER-retained missense variants also exhibited decreased plasma membrane localization, with the only exception being p.Asp527Glu (Figure 4). The protein trafficking data for p.Phe549Cys are in agreement with findings from Xie *et al.* (14), who reported that this variant demonstrated an inability to be fully N-linked glycosylated, which would therefore lead to apparent retention by the ER. However, these authors also observed complete N-linked glycosylation of p.Pro75Gln (14), which is inconsistent with the ER retention that was evident for that missense variant in this study. An explanation for this disparity may arise from their fusion of GFP to the N-terminus of PTCHD1, which, given its large size relative to the 3xFlag epitope tag used in this study, may have an intrinsic stabilizing effect on certain missense variants. Functionally, the interaction between Snapin and the first lumenal loop of Ptchd1 was not abolished by any of the 9 clinical point mutations evaluated in this study (Figure 2). This implies that the Snapin-binding domain of Ptchd1 is not structurally reliant on these specific amino acid residues. Clinically, the findings from this study suggest that the pathoetiologies of the missense variants evaluated in this study are unrelated to their interactions with SNAPIN.

A constraint inherent to the use of HEK293T cells as a model for protein expression in the context of ASD and ID is that it is non-neuronal, which may affect the neuropathological translatability of these findings. This concern is minimized by the fact that mechanisms of retention of misfolded proteins by the rough ER are highly conserved across cell types and that this system has previously been used to evaluate the subcellular localization of PTCHD1 missense variants (8). To overcome this limitation, we also sought to evaluate PTCHD1 subcellular trafficking in a neuronal context in this study. In neurons derived from differentiated N2a cells, WT PTCHD1 is trafficked to plasma membranes of both the cell body and dendrites and also displays a punctate localization pattern within dendrites (Figure 5). Of the subset of missense variants similarly assayed in this study, both p.Ser51Asn and p.Gln102Arg were appropriately trafficked to the plasma membrane of differentiated N2a cells, while p.Pro32Arg, p.Pro75Gln, p.Val150Met, p.Tyr213Cys, p.Gly303Arg, and p.Phe549Cys all qualitatively showed an inability to localize to the plasma membrane (Figure 5 and Table 2). With the exception of p.Val150Met, these data are consistent with our observations from HEK293T cells (Figure 4) or as reported by our group previously (14). Interestingly, p.Val195Ile demonstrated plasma membrane localization in our neuronal system but exhibited ER retention and diminished membrane trafficking in HEK293T cells (Figures 3 and 4). This discrepancy may suggest that neuronal cells possess a unique mechanism to allow some point mutants, despite relative instability, to exit the ER and be trafficked to their proper cellular destination.

The subcellular localization data from the current study offer a pathoetiological mechanism for a number of these missense variants, in which protein misfolding-mediated aggregation and retention in the rough ER and subsequent impaired dendritic membrane trafficking lead to insufficient bioavailable PTCHD1 at the PSD. It has also been suggested that chronic and excessive ER stress is associated with ASD pathology, potentially through impaired membrane trafficking of GABA (gamma-aminobutyric acid) receptor subunits (31) and constitutive activation of mTOR (mechanistic target of rapamycin) mediated by tuberous sclerosis complex loss of function (32). The mechanism of pathogenicity for the other missense variants evaluated in this study that showed canonical subcellular localization (p.Ser51Asn, p.Leu73Phe, p.Gln102Arg, p.Val150Met, p.Lys181Thr, and p.Thr602Ala) remains elusive. Loop regions of transmembrane proteins constitute binding pockets for interacting proteins and other biomarkers (33), and therefore it is feasible that the binding of either cholesterol or a still-unidentified PTCHD1 interacting partner may be affected by these point mutations. Collectively, these findings provide a platform for future diagnostic assays and improved interpretation of PTCHD1 VUSs by genetic diagnosticians.

### Conclusions

This study has identified and validated a novel PTCHD1 interacting partner, SNAPIN, and confirmed that selected clinical missense variants within the lumenal loops do not abolish this interaction. In addition, we report that several missense variants exhibited ER retention and impaired plasma membrane localization in neurons. Future research should be aimed at clarifying the effects of PTCHD1 on SNAPIN-mediated synaptic vesicle exocytosis; further elucidating the endogenous network of proteins interacting with PTCHD1, possibly through biotin identification; and quantifying the stability of PTCHD1 missense variants using highly sensitive biochemical techniques, such as circular dichroism, differential scanning calorimetry, or microscale thermophoresis.

## Supplementary Material

Supplement Methods, Figures S1–S5, and Tables S1–S5

Key Resource Table
