# Supplementary material for: Autism-Associated PTCHD1 Missense Variants Bind to the SNARE-Associated Protein SNAPIN but Exhibit Impaired Subcellular Trafficking
Source: Biol Psychiatry Glob Open Sci. 2025 Mar 22;5(4):100492. doi: 10.1016/j.bpsgos.2025.100492 (PMC12138416; doi:10.1016/j.bpsgos.2025.100492)
Supplement: Supplement Methods, Figures S1–S5, and Tables S1–S5 [file mmc1.pdf]

SUPPLEMENTARY INFORMATION

Autism-Associated *PTCHD1* Missense Variants Bind to the SNARE-Associated Protein, SNAPIN, but Exhibit Impaired Subcellular Trafficking

Pastore *et al.*

Contents:

Additional Methods: Yeast Two-Hybrid Screen, Cloning, Cell Culture and Neuronal Differentiation, Co-immunoprecipitation, Immunoblotting, and Immunocytochemistry

Figure S1 and S2: Sanger sequence validation of PTCHD1 missense constructs generated using site-directed mutagenesis.

Figure S3: Pedigree and Sanger validation of Q102R variant in multiplex family from Pakistan.

Figure S4: Pedigree and Sanger validation of V150M variant in multiplex family from Pakistan.

Figure S5: Clustal 2.1 alignment of PTCHD1 across vertebrate evolution, showing conservation at missense variants.

Table S1: Full results from PTCHD1 yeast two-hybrid screen.

Table S2: *In silico* predictions for pathogenicity of PTCHD1 missense variants.

Table S3. Oligonucleotides used to generate expression constructs.

Table S4. Location and clinical information for PTCHD1 missense variants.

Table S5. Oligonucleotides used for site-directed mutagenesis to generate missense variants in *Ptchd1*.

Supplementary References

Additional Methods

Yeast Two-Hybrid Screen

The Matchmaker Gold Yeast Two-Hybrid System (Takara Bio; Kusatsu, Japan) was used. Briefly, lumenal loop 1 (amino acids p.Glu49-p.Arg270) or a fusion of lumenal loop 1 to lumenal loop 2 (amino acids p.Gln521-p.Ser695) of the human orthologue of *PTCHD1* was cloned into the pGBKT7 vector in-frame with the DNA-binding domain of the Gal4 transcription factor. Constructs were separately transformed into the *S. cerevisiae* strain Y2HGold and independently used as bait to probe two separate cDNA libraries fused to the activation domain of Gal4: 1) Mate & Plate Library Mouse Embryo Day 11, and 2) Normalized Mate & Plate Library Adult Human Brain (Takara Bio; Kusatsu, Japan). Three independent screens were performed for each library. Positive clones were selected on synthetic dropout medium in the absence of tryptophan, leucine, histidine, and adenine, and supplemented with X-gal. Positive clones were subjected to plasmid DNA extraction and transformation into *E. coli*, and plasmids from individual colonies were then amplified by PCR and Sanger sequencing was used to identify the insert.

Cloning

The complete coding sequences of the mouse orthologues of *Ptchd1* and the SNARE-associated protein *Snapin*, which exhibit 98.1% and 97.8% protein sequence homology, respectively, with their human orthologues, were separately cloned into the expression vector pcDNA3.1 myc-His B (Thermo Fisher Scientific; Waltham, MA). *Snapin* was cloned in-frame with the downstream myc-His tags. For *Ptchd1*, the stop codon was included, and a 3xFlag epitope tag was subsequently inserted at the N-terminus. Finally, the coding sequences of 3xFlag-tagged *Ptchd1* lumenal loop 1 (amino acids p.Val48-p.Arg266), lumenal loop 2 (amino acid p.Tyr499-p.Ala698), and a lumenal loop 1-loop 2 chimeric protein were cloned into pcDNA3.1 myc-His B, succeeded by a stop codon. The human orthologue of *PTCHD1* was recombined into the vector pDEST53 (Thermo Fisher Scientific) in-frame with GFP via Gateway cloning, and human SNAPIN cloned into pcDNA3.1 myc-His B. Primer sequences used for expression cloning are provided in Supplementary Table S3.

Site-directed mutagenesis was employed to generate the clinically-reported *Ptchd1* missense variants evaluated in this study (Table 1). The amino acid residues for all 14 missense variants evaluated are conserved between human and mouse (see Supplementary Figure S5). Briefly, the wildtype *3xFlag-Ptchd1-pcDNA3.1* vector was amplified using Q5 high-fidelity DNA polymerase (NEB; Ipswich, MA), with the forward primer containing the mutant codon of interest. Next, purified PCR products were phosphorylated and re-circularized with DNA ligase. All mutant constructs were confirmed by Sanger sequencing (Supplemental figures S1 and S2). Primer sequences used for site-directed mutagenesis are provided in Supplementary Table S4.

Cell Culture and Neuronal Differentiation

HEK293T cells (ATCC; CRL-3216) were maintained in DMEM supplemented with 10% FBS (Wisent; Saint-Jean-Baptiste, QC, Canada) and 1% PS (Wisent). Cells were passaged every 48-72 hours at semi-confluence by trypsinization (Wisent). P19 mouse embryonal carcinoma cells (ATCC; CRL-1825) were maintained in α-MEM supplemented with 7.5% newborn calf serum (Thermo Fisher Scientific), 2.5% FBS, and 1% PS. Undifferentiated cells were passaged every 48-72 hours at semi-confluence. For immunocytochemical staining, 24 hours prior to neuronal differentiation, Lipofectamine 3000 (Thermo Fisher Scientific) was utilized according to the manufacturer's instructions to co-transfect *GFP-Ptchd1* and *Snapin-myc* into the cells. To induce neuronal differentiation, undifferentiated cells were grown on non-adherent bacterial-grade plates (Thermo Fisher Scientific) supplemented with 1 µM all-*trans* RA (Cell Signaling) for four days. On the fourth day, aggregates were plated onto sterile coverslips in 12-well plates, and allowed to differentiate for six days. Mouse Neuro-2a (N2a) neuroblastoma cells were seeded at a density of 70% confluency on coverslips in 6-well plates. They were cultured in Dulbecco's Modified Eagle Medium (DMEM) with high glucose (4.5 g/L) supplemented with 10% (*v*/*v*) fetal bovine serum (FBS, Gibco) and containing 1% (*v*/*v*) antibiotics (100 U/mL penicillin, 100 mg/mL streptomycin; Sigma-Aldrich, St Louis, MO). For transfections, DNA constructs constructs (WT or mutant N-terminal-tagged *3xFlag-Ptchd1* plus *Snapin-myc* (see above)) were mixed with 0.1% polyethylenimine (PEI; 1µgDNA:3µl PEI) (1)

and added to N2A cells. Subsequently, neuronal differentiation of N2A cells was induced by switching media to 4% FBS and adding retinoic acid to a final concentration of 2μM.

Co-Immunoprecipitation

HEK293T cells were re-seeded in 6-well culture plates. 24 hours later, Lipofectamine 3000 (Thermo Fisher Scientific) was utilized according to the manufacturer's instructions to co-transfect 2 μg of *3xFlag-Ptchd1* and 500 ng of *Snapin-myc* expression plasmids. 48 hours later, cells were washed with ice-cold PBS and lysed in immunoprecipitation (IP) buffer (50 mM Tris-HCl, pH 8.0, 150 mM NaCl, 1% NP-40) supplemented with cOmplete™ Mini EDTA-free protease inhibitors (MilliporeSigma, Burlington, MA). Lysates were incubated for 10 minutes on ice, followed by centrifugation at 10,000 x *g* for 10 minutes at 4°C. Supernatants were quantified using the Bradford total protein assay (Bio-Rad; Hercules, CA). For co-IP experiments, 500 μg of lysates were incubated with 1 μg of mouse α-Flag M2 antibody (Sigma-Aldrich; St. Louis, MO) in a final volume of 1 mL of IP buffer overnight at 4°C with rotation. The next day, lysates were incubated with 50 μl of Dynabeads Protein G (Thermo Fisher Scientific) for two hours at room temperature with rotation. Antibody-protein complexes were then separated magnetically, washed three times with PBS, and eluted from the Dynabeads using elution buffer (75 mM Glycine-HCl, pH 2.7). Co-transfected lysates were all immunoprecipitated using a mouse anti-Flag antibody, and then subsequent immunoblots were probed for Flag using a rabbit anti-Flag antibody, as indicated in Supplementary Table S5. Two biological replicates were performed for co-IP experiments.

Immunoblotting

Input lysates (5 μg of total protein) and entire elution products were combined with 4x Laemmli buffer (Bio-Rad) supplemented with 10% β-mercaptoethanol (Sigma-Aldrich, St Louis, MO). Samples were subsequently incubated at 37°C for 20 minutes to avoid boiling-induced aggregation of multi-pass transmembrane proteins (2). Samples were separated using 4-15% gradient SDS-PAGE gels (Bio-Rad), transferred to PVDF membranes (Bio-Rad), and blocked in 3% skim milk powder (BioShop) for one hour at room temperature with agitation. Blots were incubated with primary antibodies at the appropriate

concentrations in blocking solution overnight at 4°C with agitation. The next day, blots were washed three times with wash buffer (TBS supplemented with 0.2% Tween-20) and incubated with horseradish peroxidase-conjugated secondary antibodies at the appropriate concentrations in blocking solution for one hour at room temperature with agitation. Blots were then washed three additional times with wash buffer, followed by chemiluminescence detection (Thermo Fisher Scientific) using the ChemiDoc imaging system (Bio-Rad). Antibody concentrations are provided in Supplementary Table S5.

Immunocytochemistry

Sterile 13 mm coverslips (Sarstedt) were inserted into 24-well culture plates and coated with 100 µg/mL poly-D-lysine solution (Sigma-Aldrich) according to the manufacturer's instructions. HEK293T cells were re-seeded at low dilutions onto coated coverslips. The next day, Lipofectamine 3000 was utilized according to the manufacturer's instructions to co-transfect 10 ng of *3xFlag-Ptchd1* expression plasmids and 490 ng of the inert carrier plasmid pBV-Luc (www.addgene.org #16539). 24 hours later, cells were rinsed with PBS and fixed with ice-cold 100% methanol at -20°C for 15 minutes. Cells were then washed twice with PBS and incubated with blocking solution [10% goat serum (Cell Signaling) in PBS supplemented with 0.1% Tween-20] for one hour at room temperature with agitation. After blocking, cells were incubated with primary antibodies at the appropriate concentrations in blocking solution overnight in a humidified chamber at 4°C with agitation. The next day, cells were washed three times with PBS, and incubated with Alexa Fluor-conjugated secondary antibodies at the appropriate concentrations in blocking solution for one hour at room temperature with agitation in the dark. Cells were then washed three additional times with wash buffer, followed by incubation with NucBlue reagent (Thermo Fisher Scientific) according to the manufacturer's instructions. Finally, cells were mounted on glass slides using Dako mounting medium (Agilent). Antibody concentrations are provided in Supplementary Table S5. Three biological replicates were performed for immunocytochemistry experiments. For transfected N2a cells, cells grown on coverslips were fixed in 4% PFA at room temperature for 10 minutes, permeabilized with 0.1% TritonX-100 in PBS, followed by blocking with 0.5% BSA in PBS for 30 minutes at RT. The

cells were incubated with primary antibodies in 0.5% BSA in PBS solution for 1hr at room temperature or overnight at 4ºC. After washing 3 times with 0.1% TritonX-100 in PBS, the cells were then incubated with AlexaFluor secondary antibodies for 45 minutes at room temperature. The cells were then washed washing 3 times with 0.1% TritonX-100 in PBS and then mounted onto slides using VectaShield Mounting media with DAPI (Vector Labs, #64335). Images were taken using the Nikon Eclipse 80*i* fluorescence microscope using QICAM-UV Fast 1394 camera (QImaging, BC, Canada) and QCapture Suite PLUS software.

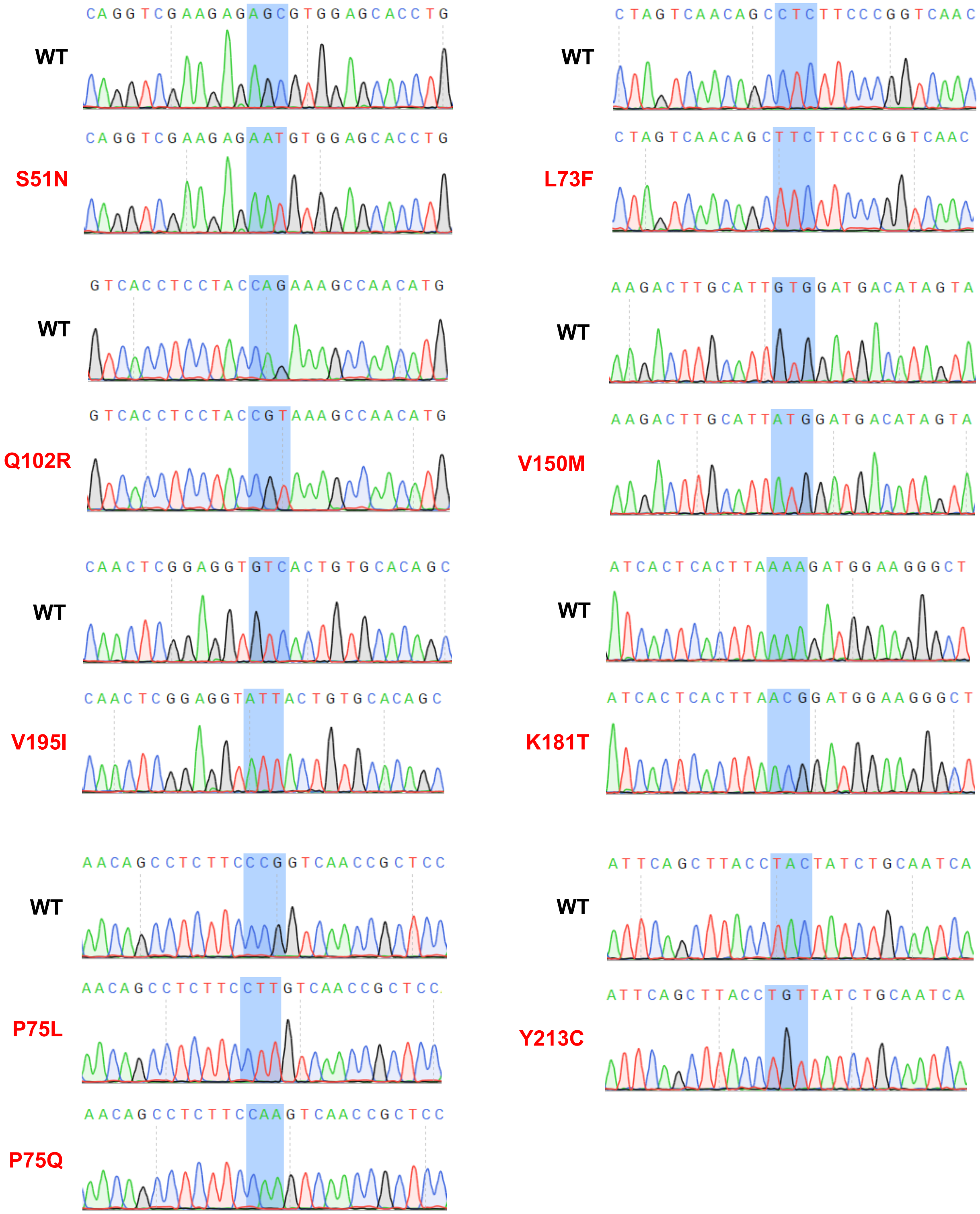

Figure S1. DNA electropherograms of lumenal loop 1 missense variants.

Mutant codons are highlighted in blue. Missense variants p.Ser51Asn, p.Leu73Phe, p.Pro75Leu, p.Pro75Gln, p.Gln102Arg, p.Val150Met, p.Lys181Thr, p.Val195Ile, and p.Tyr213Cys are located in lumenal loop 1

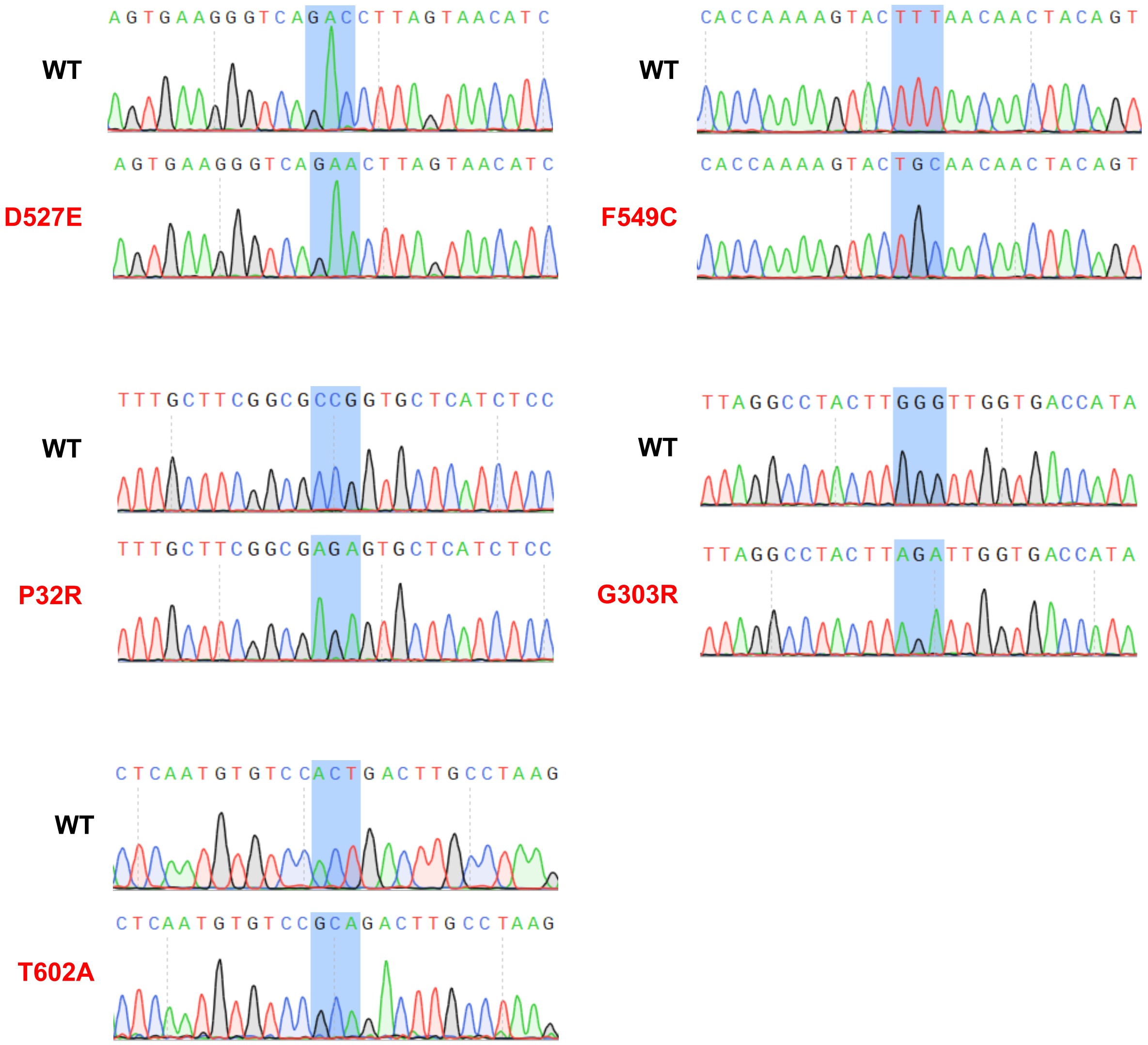

Figure S2. DNA electropherograms of lumenal loop 2 and TMD missense variants.

Mutant codons are highlighted in blue. Missense variants p.Pro32Arg and p.Gly303Arg are located in the first and third TMDs, respectively; missense variants p.Asp527Glu, p.Phe549Cys, and p.Thr602Ala are located in lumenal loop 2

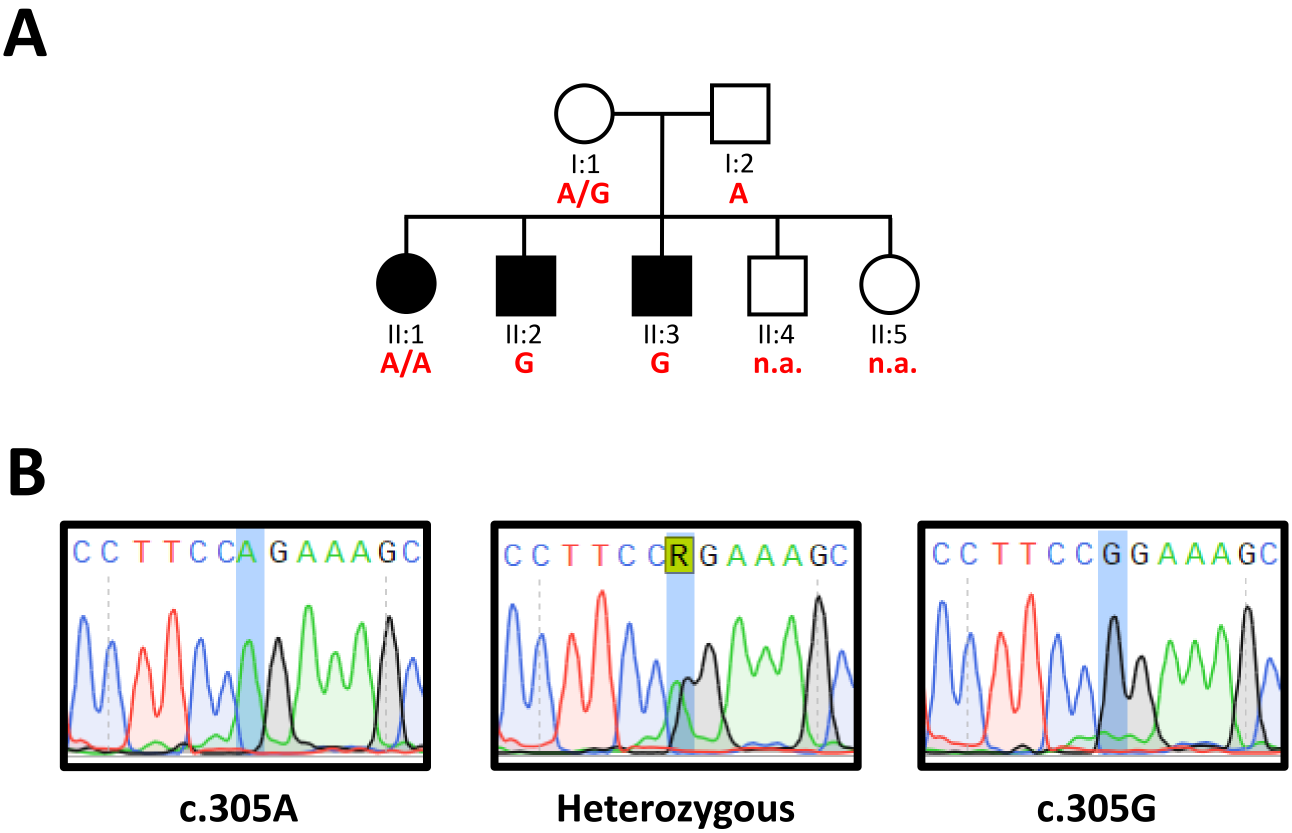

Figure S3. SNV Identification (c.305A>G; p.Gln102Arg) in a multiplex ID Pakistani family.

A) Pedigree of Family ARSID_M4 with two males and one female diagnosed with ID. ARSID_M4 was involved in a whole exome sequencing study by our group, as yet unpublished. Both affected males (II:2 and II:3) contain a point mutation (c.305A>G; p.Gln102Arg), which was inherited from their unaffected mother (I:1). **B)** Electropherograms showing the canonical *PTCHD1* sequence *(top)*, the c.305A>G point mutation *(bottom)*, and the heterozygous maternal carrier *(middle)*.

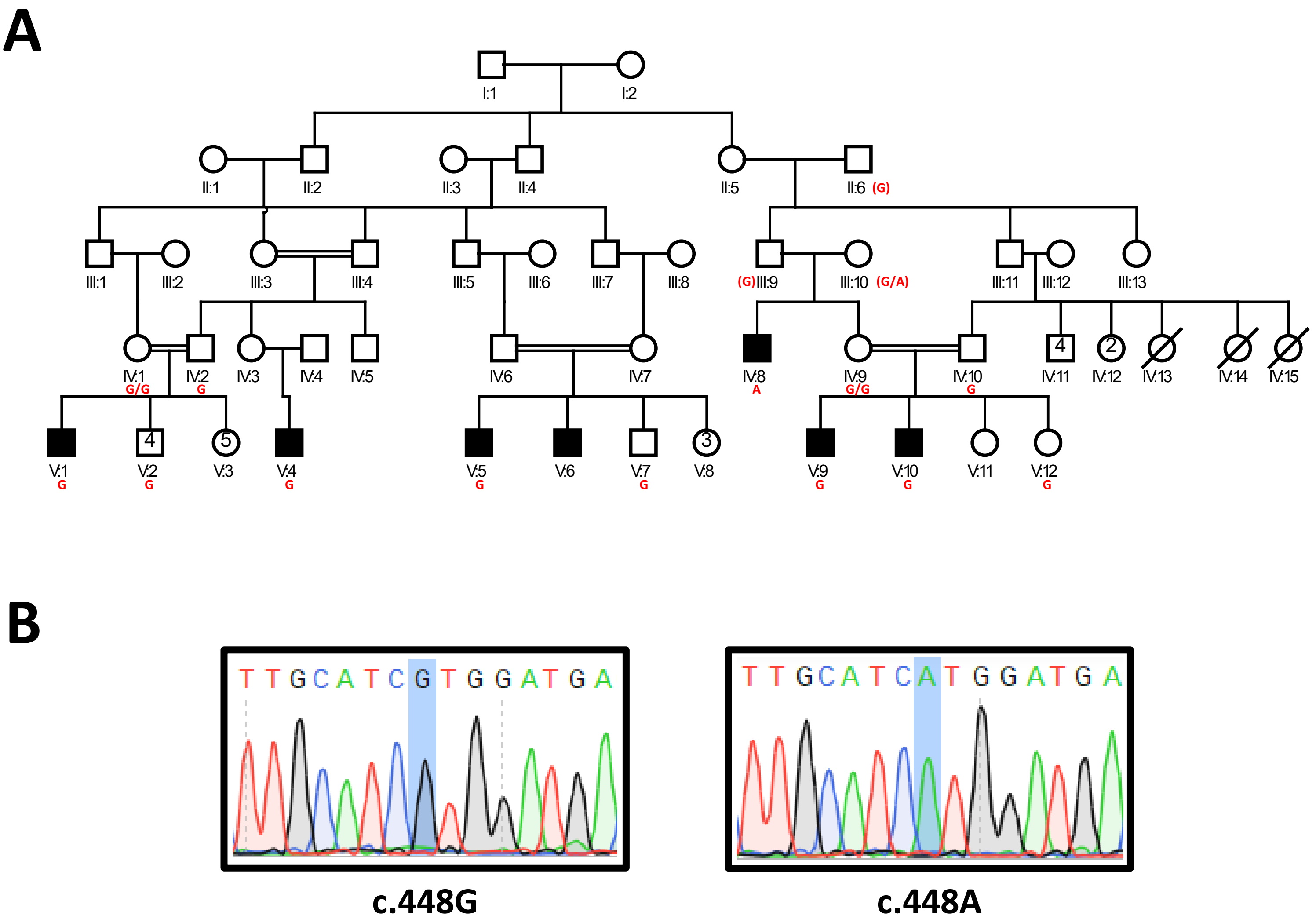

Figure S4. SNV Identification (c.448G>A; p.Val150Met) in a multiplex ID Pakistani family.

A) Pedigree of Family AS30 with seven males diagnosed with ID. AS30 was involved in a whole exome sequencing study, reported in Harripaul et al, 2018 (PMID 28397838), however this variant was not listed. One of these affected males (IV:8) contains a point mutation (c.448G > A; p.Val150Met), which is *de novo* or was inherited from his unaffected mother (III:10). **B)** Electropherograms showing the canonical *PTCHD1* sequence *(top)* and the c.448G > A point mutation *(bottom)*.

Figure S5. Evolutionary sequence alignments for PTCHD1 using CLUSTAL 2.1. Protein sequences from representative species spanning >400 million years of the vertebrate lineage, including: human (NP_775766.2), mouse (NP_001087219.1), Tasmanian devil (XP_003763547.1), alligator (XP_006266959.1), Xenopus (XP_004911719.1), zebrafish (XP_690754.1), coelacanth (XP_006013422.1), and lamprey (Ensembl gene prediction ENSPMAT00000029719.1). Residues corresponding to missense variants evaluated in this study are indicated with red highlight.

```
                                                P32R                S51N
human           MLRQVLHRGLRTCFSRLGHFIASHPVFFASAPVLISILLGASFSRYQVEESVEHLLAPQH
mouse           MLRQVLHRGLRTCFSRLGHFIASHPVFFASAPVLISILLGASFSRYQVEESVEHLLAPQH
Tas_devil       MLRQVLHRGLRTSFSRLGHFIASHPVFFASAPVLISILLGASFSRYQIEESVEHLLAPKH
alligator       MLRQVLHRGLRTSFSRLGHFVASHPVFFASAPVLISILLGASFSRYHIEESVESLLAPKH
canary          MLRQVLHRGLGTSFSRLGHFVASHPVFFASAPVLISILLGASFSRYQVEESVEHLLAPTH
xenopus         MLRKVLHRGLRNCFSRLGFFIASHPVFFISAPVLISILLGASFSRYRVEENIEYLLAPKH
coelacanth      MLRQVLHKGLRTCFSRLGYFIASHPVFFASAPVLISILLGASFSRYRIEENVEYLLAPKH
zebrafish       MLRQVLHEGLRTSFHKLGHFVANHPVFFASAPVLISILLGASFSRYRIEENVEYLLAPKH
lamprey         MLGALLHAALQRCLYRLGLLVAGHPAPFLVAPALLAALLGAALSRVSVESKAEDLFAPAH
                         L73F P75Q/L                 Q102R
human           SLAKIERNLVNSLFPVNRSKHRLYSDLQTPGRYGRVIVTSFQKANMLDQHHTDLILKLHA
mouse           SLAKIERNLVNSLFPVNRSKHRLYSDLQTPGRYGRVIVTSYQKANMLDQHHTDLILKLHT
Tas_devil       SLAKIERNLVNSLFPVNRSKHRLYSDLQTPGRYGRVIITSFQQENMLDQHHTDLILKLHS
alligator       SLAKIERNLVNSLFPVNRSKHRLYSDLQTPGRYGRVIITSFRKANMLDQHHTELILKLHS
canary          SLAKIERNLVDSLFPVNRSKHRLYSDLQTPGRYGRVIITSFRKANMLDQHHTDLILKLHS
xenopus         SLAKIERNLVDSLFPVNRSKHRLYSDLQTPGRYGRVIITSSRRANMLDQHHTDLILKLHS
coelacanth      SLAKIERNLVDSLFPVNRSKHRLYSDLQTPGRYGRVIITSSRKGNMLDQFHTDLILKLHF
zebrafish       SLAKIEGNLVDSLFPVNRSKHTLYSDLQTPGRYGRVIVTS-RRGSVLDPHHVNSVLKLHN
lamprey         SLAKLEGALADELFPLERSRQRLYSELHTPGRYTRLIAVARGGGNVLDEGRRLALISLHS
                            V150M
human           AVTKIQVPRPGFNYTFAHICILNNDKTCIVDDIVHVLEELKNARATN---RTNFAITYPI
mouse           AVTKIQVPRPGFNYTFAHICVLNNDKTCIVDDIVHVLEELKNARATN---RTNFAITYPI
Tas_devil       AVTRMQVQRPGFNYTFAHICILNNDKTCIVDDIVHVLEELKTARATN---RTNFAITYPI
alligator       AVTRIQVQRPGFNYTFAHICILNNDKTCIVDDIVHVLEELKTARSSN---RTNFAITYPI
canary          AVTRIQVQRPGFNYTFAHICILNNDKTCIVDDIVHVLEELKAARSSN---RTNFAITYPI
xenopus         AVKKIQVHRPGFNYTFAHICMLSNEKTCIVDDIVHILEELKAARSQN---RTNYIITYPI
coelacanth      SVNKIQVPMLGINYTFAHMCVLNDDKTCIVDDVVHILEELQASRSLN---KTGFTIMYPI
zebrafish       TITQIQVPMLGFNYTFAHLCLLDESKSCIVDDILRVLEEMRSARASN---HSVPPLRYPI
lamprey         ALVAMPVGR---NCTFWDVCSIDWDRECVQDAIVELMGNGSSSASDASRRRSSIIIRYPK
                 K181T       V195I               Y213C
human           THLKDGRAVYNGHQLGGVTVHS-KDRVKSAEAIQLTYYLQSINSLNDMVAERWESSFCDT
mouse           THLKDGRAVYNGHQLGGVTVHS-KDRVKSAEAIQLTYYLQSINSLNDMVAERWESSFCDT
Tas_devil       THLKDGREVYNGHQLGGVTVHS-KDQVKSAQAVQLTYYLQTLNSLNDMVAERWESNFCDT
alligator       THLKDGREVYNGHQLGGVTVHS-KDRVKSAEAIQLTYYLQAINSLNDMVAEKWESIFCNT
canary          THLKDGREVYNGHQLGGVTVHS-KDRVKSAEAIQLTYYLQAINSLNDMVAEKWESIFCDT
xenopus         TKLKDGKEVYNGHQLGGVTVHS-KDRVKSAEAIQLTYYLQAINSLNEVVAEKWESVFCET
coelacanth      THLKNDREVYIGHQLGGVTLHS-KDRVKSAEAIQLTYYLQTINFLNDMVAEKWESIFYET
zebrafish       TKLKDGREAYIGHQLGGVLASGGRDGVRSARALQLTYYLQAVSPLNEVVAASWELLFCRE
lamprey         ARLRDNQEVYIGHQLGGVTLFQ-TDRVLSARALQITYYLDSAHAA----SELWEREFVTA

human           VRLFQK-SNSKVKMYPYTSSSLREDFQKTSRVSERYLVTSLILVVTMAILCCS-MQDCVR
mouse           VKLFQK-SNSKVKIYPYTSSSLREDFQKTSRVSERYLVTSLILVVTMAILCCS-MQDCVR
Tas_devil       VKLFQK-SNGKIKMYPYTSSTLREDFQKTSRVSERYLITSLVLMVTMAVLCCS-MQDCVR
alligator       VELFQK-SNRKVKMYPFTSSSLKEDFQKTSRVSERYLITSLVLVVSLAILCCS-MQDCVR
```

```
canary        VELFQK-SNRKVKMYPFTSSSLKEDFQKTSRVSERYLITSLVLVVTLAILCCS-MQDCVR
xenopus       VENFQK-LNREVKLYPFTSSSLGQDFQKTSRVSERYLITSLALVVSLAVICCS-MQDCVR
coelacanth    VELFQA-SNGAVKLYPFTSSSLSEDFQKTSRVSEQYLITSLILVVFLAILCCS-MRDCVR
zebrafish     LENFGK-AHPELSLHPFTSSSLQRDFQRTSRVSERPLLFSLAVCLSLAMLCCS-MRDCVR
lamprey       VERARHRHAHDLALFPFTSSSLQTDFYQSGVVAAPNLAGGCGGWWTALKNSRGRTRSEDL
                      G303R
human         SKPWLGLLGLVTISLATLTAAGIINLTGGKYNSTFLGVPFVMLGHGLYGTFEMLSSWRKT
mouse         SKPWLGLLGLVTISLATLTAAGIINLTGGKYNSTFLGVPFVMLGHGLYGTFEMLSSWRKT
Tas_devil     SKPWLGLLGLVTVSLATLTAAGIINLTGGKYNSTFLGVPFVMLGHGLYGTFEMLSSWRKT
alligator     SKPWLGLLGLLTISLATLTAAGIINLTGGKYNSTFLGIPFIMLGHGLYGTFEMLSSWRKT
canary        SKPWLGLLGLLTVTLATLTAAGIINLTGGKYNSTFLGIPFVMLGHGLYGTFEMLSSWRKT
xenopus       SKPWLGLLGLVTISLSTLTAAGIINLTGGKYNSTFLGIPFIMLGHGLYGTFEMLSSWRKT
coelacanth    SKPWLGILGLVTMSLATLTSAGIINLTGGKYNSTFLGIPFIVLGHGLYGTFEMLSSWRKT
zebrafish     TKPWLGLLALVTVSLATLTSAGILNLTGGKYNSTYLGIPFVMLGHGLFGTFEMLSSWRRT
lamprey       CFGLLVLAGFVGIALTTLATAGVMILTGTPYNSTLIIIPLVALGHGSHGATELLWTWRRL

human         RE-----DQHVKERTAAVYADSMLSFSLTTAMYLVTFGIGASPFTNIEAARIFCCNSCIA
mouse         RE-----DQHVKERTAEVYADSMLSFSLTTAMYLVTFGIGASPFTNIEAARIFCCNSCIA
Tas_devil     RE-----DQHVKERTAAVYADSMLSFSLTTAMYLVTFGIGASPFTNIEAARIFCCNSCIA
alligator     RE-----DQHVKERTAAVFADSMLSFSLTTAMYLVTFGIGASPFTNIEAARIFCCNSCIA
canary        RE-----DQHVKERTAAVFADSMLSFSLTTAMYLVTFGIGASPFTNIEAARIFCCNSCIA
xenopus       RE-----DQHVKERTAVVYADTMISFTLTTAMYLVTFGIGASPFTNIEAARIFCRNSCIA
coelacanth    RE-----DQHVKERMATVYADSMLPFSFTTAMYLVTFGIGASPFTNIEAARIFCRNSCIA
zebrafish     RE-----DQHVKERVAAVFSDCMLPFTASTALHVVTFGIGASPFTNIEAVRLFCQNACIS
lamprey       GWGARGPPAREEERLAATFARSLLPHTMLTALQVITLALGASPLTNTRAVQVFCRCACTA

human         IFFNYLYVLSFYGSSLVFTGYIENNYQHSIFCRKVPKPEALQEKPAWYRFLLTARFSEDT
mouse         ILFNYLYVLSFYGSSLVFTGYIENNYQHSIFCRKVPKPDVLQEKPAWYRFLLTARFSEET
Tas_devil     IFFNYLYVLSFYGSSLVFTGYIENNYQHSLFCRKVPKPEVLQEKPAWYRFLLTARFSEDT
alligator     IFFNYLYVLSFYGSSLVFTGYIENNYQHSIFCRKVPKPEVLQEKPAWYRFLLTAKFSEDT
canary        IFFNYLYVLSFYGSSLVFTGYIENNYQHSIFCRKVPKPEVLQEKPAWYRFLLTARFSEDT
xenopus       IFFNYLYVLSFYGSSLVFTGYIENNYQHSIFCRKVPKPEILQVKSLWYRFLMTAKFSDDT
coelacanth    VFFNYLYVLSFYGSNLVFTGYMENNYQHSLFCRKVPKPEVLQGKPLWYRLLLTAKYNEET
zebrafish     VLFNYLYILTFYGSNLVFAGYLENNYRHSLFCRRVPKPELLQQKPAWYRFLMYTHYNEEA
lamprey       TALGYCACVTFLGASSDVTPRGLQDLKTPRCICEQPDLISSKEPPCSTATTNTTNTASTA

human         AEGEEANTYES--------HLLVCFLKRYYCDWITNTYVKPFVVLFYLIYISFALMGYLQ
mouse         AEGEEANTYES--------HLLVCFLKRYYCDWITNTYVKPFVVLFYLIYISFALMGYLQ
Tas_devil     ADGEEANTYES--------HLLVCFLKRYYCDWITNTYVKPFVVLFYLIYISFALMGYLQ
alligator     TDSEETNTYES--------HLLVCFLKRYYCDWITNTYVKPFVVLFYLVYISFALMGYLQ
canary        DDSEETNTYES--------HLLVWFLKRYYCDWITNTYVKPFVVLFYLVYISFALMGYLQ
xenopus       ADAEETNSYES--------HLLVCFLKRYYCDWITNTYVKPFVILFYLVYISFALMGYLQ
coelacanth    ADSVETSTYES--------HLLICFLKRYYCDWITNTYVKPFVVLFYLVYISFALMGYLQ
zebrafish     TEAGPLCAYES--------HLLVAFMKRYYCDWITNTYVKPFVVLFYLVYVSFALMGYLQ
lamprey       AVTTTTTAAAATSGPHPPDAPPRRFMRDRYGPWISGTYVKPFVVLLYLVYASFSFMGCLQ
                   D527E                 F549C
human         VSEGSDLSNIVATATQTIEYTTAQQKYFSNYSPVIGFYIYESIEYWNTSVQEDVLEYTKG
mouse         VSEGSDLSNIVATATQTIEYTTAHQKYFNNYSPVIGFYIYESIEYWNSSVQEDVLEYTKG
Tas_devil     VREGSDLSNIVATATRTIEFTTAQQKYFSNYSPVIGFYIYESIEYWNTSVQEDVLEYTKG
alligator     VNEGSDLSNIVATATRTIEYTTAQQKYFSNYSPVIGFYIYESIEYWNTSVQEDVLEYTKG
canary        VSEGSDLSNIVATATRTIEYTTAQQKYFSNYSPVIGFYIYESIEYWNTSVQEDVLEYTKG
xenopus       VHEGSDLRNIVATETRTITYTTVQQKYFSNYSPVIGFYIYESIDYWNTSVQEDVLEYTKG
coelacanth    VNEGSDLSNIVATETRTIAYTTAQQKYFSNYSPVIGFYIYESIEYWNISVQEDVLEYTKG
zebrafish     VSEGSDLSNVVATETSTIAYTRAQQRYFSSYSPVIGFYIYESIEYWNTSVQEDLLEYIKG
lamprey       LREASDLTHLVASRSATARYRAVQDRFFSDYSPVIGFYIYEPVAYWNASVQEDLLDITRR
```

```
                         T602A
human        FVRISWFESYLNYLRKLNVSTGLPKKNFTDMLRNSFLKAPQFSHFQEDIIFSKKYNDEVD
mouse        FVRISWFESYLNYLRKLNVSTDLPKKNFTDMLRNSFLKTPQFSHFQEDIIFSKKYNDEVD
Tas_devil    FVRISWFESYLNYLRKLNASTGLPKKNFTDMLRNSFLKAPQFSHFSEDIIFSKKFNNEVD
alligator    FVRISWFESYLNYLRKLNISTGLPKKNFTDMLRNSFLKAPQFAHFSEDIIFSKKYNNEVD
canary       FVRISWFESYLNYLRKLNISTGLPKKNFTDMLRNSFLKTPQFAHFSEDIIFSKKYNNEVD
xenopus      FVRISWFESYLNYLRKLNMSTGLPKKNFTDILRYSFLKNPQYAHFSEDIIIPKKYNNDVE
coelacanth   FVRISWFESYLNYLRKLNMTTGLPKKNFTEMLRNSFLKTPQFSHFSEDIIFAKKYNNEVE
zebrafish    FERISWFESYLNYLHGLNITTSLSRSNFTERLRSGFLRQPRYVHFTDDIIFAKRSDGEFD
lamprey      FVTVSWLEQYTRYLRAANGTSALPRDLFVSTLCASFLRRREFAHFADDVVLAGP-PGERR

human        VVASRMFLVAKTMETNREELYDLLETLRRLSVTSKVKFIVFNPSFVYMDRYASSLGAPLH
mouse        VVASRMFLVAKTMETNREELYDLLETLRRLSVTSKVKFIVFNPSFVYMDRYASSLGAPLH
Tas_devil    VVASRMFLVAKTMETNREELYDLLETLRRLSVTSKVKFIVFNPSFVFMDRYSSSLGAPLQ
alligator    VVASRMFLVAKTMETKREELYDLLETLRKLSLTSKVKFIVFNPSFVYMDRYASSVGAPLQ
canary       VVASRMFLVAKTMETKREELYDLLETLRKLSLTSKVKFIVFNPSFVYMDRYASSVGAPLQ
xenopus      VVASRMFLVAKTMETNREELYDLLETLRKLSLTSKVKFIVFNPSFVYMDRYASSVGAPLQ
coelacanth   VVASRMFLVAKTMETKREELYDLLETLRKLSLTSKVKFIIFNPSFVYMDRYASSIGAPLQ
zebrafish    VVASRMFLIAKTTENKREEMSILLDTLRKLSLTSRVKFIIFNPSFVYMDRYASSVGAPLK
lamprey      IAASRVFMVAKTNENTREEIDGLLEALRKLSLTSRVKFTIHNPAFAFLERYALWVGAPAH

human        NSCISALFLLFFSAFLVAD--SLINVWITLTVVSVEFGVIGFMTLWKVELDCISVLCLIY
mouse        NSCISALFLLFFSAFLVAD--SLINVWITLTVVSVEFGVIGFMTLWKVELDCISVLCLIY
Tas_devil    NSCISALFLLFFSAFLVAD--SLINVWITLTVASVEFGVIGFMTLWKVELDCISMLCLIY
alligator    NSCISALFLLFFSAFLVAD--SLINVWLTLTVASVEFGVIGFMTLWKVELDCISVLCLIY
canary       NSCISALFLLFFSAFLVAD--SLINVWLTLTVASVEFGVIGFMTLWKVELDCISVLCLIY
xenopus      NSCISALFLLFFSAFLVAS--SIINVWITLTVASVEFGVIGFMTLWKVELDCISVLCLIY
coelacanth   NSCISGLFLLFFSAFLVAD--SLINVWLTVTVASVEFGVIGFMTLWKIELDCISVLCLIY
zebrafish    NSCIAALFLLFFSTFLAAD--PLVNAWLTVTVASVEFGLVGFMTLWRVELDCVSVLCLIY
lamprey      AAALAAALALLLSAGLAPPGAAPAHAWVALCAASVQFGVLGSLGLMGAQLDCAAVLCLVY

human        GINYTIDNCAPMLSTFVLGKDFTRTKWVKNALEVHGVAILQSYLCYIVGLIPLAAVPSNL
mouse        GINYTIDNCAPLLSTFVLGKDFTRTKWVKNALEVHGVAILQSYLCYIVGLFPLAAVPSNL
Tas_devil    GINHTIDNCAPLLSTFVLGKDFTRTKWVKNALETHGVAILQSYLCYIVGLIPLAAVPSNL
alligator    GINYTIDNCAPLLSTFVLGKDFTRTKWVKNALEIHGVAILQSYLCYIVGLIPLAAVPSNL
canary       GINYTIDNCAPLLSTFVLGKEFTRTKWVKNALEVHGVAILQSYLCYIVGLIPLAAVPSNL
xenopus      GINYTIDNCAPLVSTFILGKEFSRTKWVKNSLEVHGVAILQSYLCYTVGLIPLAAVPSNL
coelacanth   GINYTIDNCAPLISTFVLGKDFTRTKWVKNTLELHGVAILQSYLCYTVGLIPLAAVPSNL
zebrafish    GVNYAVDSSAPLVSAFALGRESTRTRWVKLALEQHGVPALQSYLCYGAALLPLAAVPSNL
lamprey      SLCYSAHACAPLVATFAMGRGKSRAHWTAAALDAHAAPLLHACLWFCAAVVALAAAPSNL

human        TCTLFRCLFLIAFVTFFHCFAILPVILTFLPPSKKKRKEKKNPE-NREEIECVEMVDIDS
mouse        TCTLFRCLFLIAFVTFFHCFAILPVILTFLPPSKKKRKEKKNPE-NREEIECVEMVDIDS
Tas_devil    TCTLFRCLFLIAFVTFFHCFAILPVILTFLPPSKKKRKEKKNPE-NREEIECVEMVDMDS
alligator    TRTLFRCLFLIALVTFFHCFAILPVILTFVPPSKKKRKEKKNPE-NREEIECVEMVDMDS
canary       TRTLFRCLFLIALVTFFHCFAILPVILTFLPPSKKKRKEKKNPE-NREEIECVEMVDMDS
xenopus      TRTLFRCLFLIAFVTFFHCFAILPVILTFVPPSKKKRKEKKTPE-NREEIECVEMVDLDS
coelacanth   TRTLFRCLFLIAFVTFFHCFAILPVILTFLPPSKKKKKEKKNPE-HREEIECVEMVDS--
zebrafish    TRTLFRCLFLTAIITAFHCLAILPVLLTFLPPSKKKRRERKNAAENREEIECVEMVDS--
lamprey      ARTVARCLGLASALSAVHCLVFLPVFLTICPPSKAVKRRRQAGDGEAEEGDAAGAAAGAT

human        TRVVDQITTV
mouse        TRVVDQITTV
Tas_devil    TRVVDQITTV
alligator    TRVVDQITTV
```

```
canary          TRVVDQITTV
xenopus         TRVVDQITTV
coelacanth      TRVVDQITTV
zebrafish       TRVVDQITTV
lamprey         DGAVDQATSV
```

Table S1. Full results from PTCHD1 yeast two-hybrid screen. Screen 1 used human PTCHD1 sequence from luminal loops 1 and 2 against an E11 mouse embryo cDNA library. Screen 2 used just PTCHD1 loop 1, against an adult human brain cDNA library.

Protein	Screen	Frequency of Hits
COX11		3
MATH1	PTCHD1^{L1-L2} vs. E11 Mouse Embryo cDNA Library	2
PAX3		1
A2M		2
ANKD49		1
ANKRD55		2
ATG5		1
CPLX2		1
DCAF17		1
EPB41L1		1
GFM2		1
HSPD1		1
KDM1A		1
KLHL32		1
METAP2	PTCHD1^{L1} vs. Adult Human Brain cDNA Library	1
NOL4		1
PDZD2		1
PON		1
PSMD14		1
SNAPIN		17
STMN2		1
SYNE1		1
TADA1		1
TTC8		1
YWHAZ		1
ZnF277		2
ZnF350		1

Table S2: *In silico* predictions for pathogenicty of PTCHD1 missense variants.

In order to predict the consequences of point mutations on protein pathogenicity, ten separate computational algorithms were employed: PROVEAN (3), SIFT (4), PPH2 (5), Condel (6), CADD (7), REVEL (8), MetaLR (9), Mutation Assessor (10), InMeRF (11), and MPC (12). Missense variants were deemed to be likely pathogenic or benign based on algorithm-specific thresholds for pathogenicity. Red and green cells indicate that the missense mutation is predicted to be likely pathogenic or benign, respectively, according to the pathogenicity threshold for the given algorithm. For the PPH2 algorithm, yellow cells indicate possible predicted missense variant pathogenicity.

Missense Variant	PROVEAN	SIFT	PPH2	Condel	CADD	REVEL	MetaLR	Mutation Assessor	InMeRF	MPC
p.Ser51Asn	0.03191	0.84	0	0.42475	21.2	0.25400	0.32900	0.08100	0.28100	0.50897
p.Leu73Phe	0.03041	0.13	0.127	0.54109	22.7	0.55500	0.57800	0.52800	0.56500	0.89012
p.Pro75Gln	0.90332	0.03	0.978	0.55643	25.9	0.84300	0.56000	0.56000	0.91700	0.88235
p.Pro75Leu	0.78046	0	1	0.55652	28.6	0.83700	0.33700	0.56000	0.56000	0.86978
p.Gln102Arg	0.17834	0.61	0	0.38084	18.6	0.26200	0.02100	0.00300	0.23100	0.56322
p.Val150Met	0.19933	0.04	0.236	0.53861	23.3	0.39500	0.69600	0.33800	0.59900	0.76398
p.Lys181Thr	0.14193	0.19	0.001	0.53828	21.9	0.28600	0.49000	0.40300	0.48200	0.61421
p.Val195Ile	0.20791	0.08	0.709	0.54120	21.8	0.62200	0.74600	0.37800	0.92200	0.63626
p.Tyr213Cys	0.96395	0	0.995	0.53725	26.5	0.91500	0.77800	0.30200	0.78800	0.89993
p.Asp527Glu	0.15578	0.15	0.752	0.53206	23.3	0.52000	0.63200	0.41100	0.85600	0.87029
p.Phe549Cys	0.94899	0	1	0.56124	28.0	0.91400	0.75500	0.56000	0.88200	0.92409
p.Thr602Ala	0.06026	1	0.996	0.48406	20.9	0.47800	0.35800	0.16000	0.86300	0.87650

Table S3. Oligonucleotides used to generate expression constructs.

Full or partial restriction sites, AUG codons, and stop codons are highlighted in yellow, green, and red, respectively. For Gateway cloning, attB1 and attB2 sequences are denoted in grey and magenta, respectively. Annealing temperatures for primer pairs are also shown.

Primer	Sequence	Annealing Temperature
BamHI-Ptchd1_F	5’-TACCGGATCCATGCTGCGGCAGGTTCTGC-3’	70°C
AgeI-Ptchd1_R	5’-TACCACCGGTTCACACTGTGGTTATTTGGTCAACCAC-3’	
BamHI-Loop 1_F	5’-TACCGGATCCATGGTCGAAGAGAGCGTGGAGCAC-3’	72°C
AgeI-Loop 1_R	5’-TACCACCGGTTCAACGGCTGGTCTTCTGGAAATCTTCC-3’	
BamHI-Loop 2_F	5’-TACCGGATCCATGTATGTCAAGCCTTTTGTAGTCCTCTTTTACC-3’	69°C
AgeI-Loop 2_R	5’-TACCACCGGTTCAGGCTCCCAGAGAGGAGGC-3’	
NheI-Loop 1_R	5’-TACCGCTAGCACGGCTGGTCTTCTGGAAATCTTCC-3’	72°C
NheI-Loop 2_F	5’-TACCGCTAGCTATGTCAAGCCTTTTGTAGTCCTCTTTTACC-3’	69°C
HindIII-Snapin_F	5’-TACGTACGAAGCTTATGGCGGGGGCTGGTTCC-3’	72°C
HindIII-Snapin_F	5’-TACGTACGAAGCTTATGGCGGGGGCTGGTTCC-3’	
attB1-PTCHD1_F	5’-GGGGACAAGTTTGTACAAAAAAGCAGGCTATGCTGCGGCAGGTTC TGC-3’	72°C
attB2-PTCHD1_R	5’-GGGGACCCAGCTTTCTTGTACAAAGTGGTTCACACTGTTGTAATTT GGTCAACCACACGG-3’	
HindIII-3xFlag-Link_F	5’-AGCTTATGGACTACAAGGACCACGACGGTGACTACAAGGACCACGACATCGACTACAAGGA CGACGACGACAAGGGAGGTGGCGGAGGTTCCGGG-3’	
BamHI-3xFlag-Link_R	5’-GATCCCCGGAACCTCCGCCACCTCCCTTGTCGTCGTCGTCCTTGTAGTCGATGTCGTGGTCCT TGTAGTCACCGTCGTGGTCCTTGTAGTCCATA -3’	

Table S4. Oligonucleotides used for site-directed mutagenesis to generate missense variants in *Ptchd1*.

Mutant codons are shown in red. Annealing temperatures for primer pairs are also shown.

Location	Mutation	Sequence	Anneal Temperature
TM 1	P32R	F: 5'-AGAGTGCTCATCTCCATCCTGCTC-3'<br>R: 5'-CGCCGAAGCAAAGAAGACCG-3'	69°C
Loop 1	S51N	F: 5'-AATGTGGAGCACCTGCTGGCG-3'<br>R: 5'-CTCTTCGACCTGGTAGCGGC-3'	72°C
	L73F	F: 5'-TTCTTCCCGGTCAACCGCTCCAAG-3'<br>R: 5'-GCTGTTGACTAGGTTGCGCTCGATC-3'	72°C
	P75L	F: 5'-CTTGTCAACCGCTCCAAGCACCG-3'<br>R: 5'-GAAGAGGCTGTTGACTAGGTTGCG-3'	71°C
	P75Q	F: 5'-CAAGTCAACCGCTCCAAGCACCG-3'<br>R: 5'-GAAGAGGCTGTTGACTAGGTTGCG-3'	71°C
	Q102R	F: 5'-CGTAAAGCCAACATGCTAGACCAAC-3'<br>R: 5'-GTAGGAGGTGACAATGACCCG-3'	67°C
	V150M	F: 5'-ATGGATGACATAGTACACGTCC-3'<br>R: 5'-AATGCAAGTCTTATCATTATTCAGGAC-3'	60°C
	V195I	F: 5'-ATTACTGTGCACAGCAAGGATCGG-3'<br>R: 5'-ACCTCCGAGTTGGTGCCCATTATAC-3'	71°C
	K181T	F: 5'-ACGGATGGAAGGGCTGTGTATAATG-3'<br>R: 5'-TAAGTGAGTGATCGGATATGTG-3'	62°C
	Y213C	F: 5'-TGTTATCTGCAATCAATCAACAGTCTC-3'<br>R: 5'-GGTAAGCTGAATGGCCTCTG-3'	63°C
TM 3	G303R	F: 5'-AGATTGGTGACCATAAGCCTAGCCAC-3'<br>R: 5'-AAGTAGGCCTAACCAGGGTTTGC-3'	70°C
Loop 2	D527E	F: 5'-GAACTTAGTAACATCGTAGCAACTGCA-3'<br>R: 5'-TGACCCTTCACTGACCTG-3'	65°C
	F549C	F: 5'-TGCAACAACTACAGTCCTGTTATTGGG-3'<br>R: 5'-GTACTTTTGGTGGGCAGTAG-3'	64°C
	T602A	F: 5'-GCAGACTTGCCTAAGAAAAATTTCACAGACATG-3'<br>R: 5'-GGACACATTGAGTTTCCGAAGATAATTTAAATAGC-3'	67°C

Table S5. Antibodies and dilutions used for IP, immunoblotting, and immunocytochemistry.

Experiment	Antibody (Conjugate)	Source	Dilution
IP; Immunoblotting	Mouse α-Flag	Sigma-Aldrich #1804	1:250 (IP)
	Rabbit α-DYKDDDDK	Cell Signaling #14793	1:750
	Rabbit α-myc	Cell Signaling #2278	1:750
	Goat α-mouse (HRP)	Promega #W402B	1:2500
	Goat α-rabbit (HRP)	Promega #W401B	1:2500
Immunocytochemistry	Mouse α-Flag	Sigma-Aldrich #1804	1:300
	Rabbit α-Atla	Abcam #76020	1:500
	Rabbit α-CNX	Abcam #92573	1:1000
	Goat α-rabbit (Alexa Fluor 594)	Thermo Fisher #A11001	1:1000
	Goat α-mouse (Alexa Fluor 488)	Thermo Fisher #A11012	1:1000

Supplementary References

1. Longo PA, Kavran JM, Kim MS, Leahy DJ. Transient mammalian cell transfection with polyethylenimine (PEI). Methods Enzymol. 2013;529:227–40.
2. Fleet AJ, Hamel PA. The protein-specific activities of the transmembrane modules of Ptch1 and Ptch2 are determined by their adjacent protein domains. J Biol Chem. 2018 Oct 26;293(43):16583–95.
3. Choi Y, Chan AP. PROVEAN web server: a tool to predict the functional effect of amino acid substitutions and indels. Bioinformatics. 2015 Aug 15;31(16):2745–7.
4. Ng PC, Henikoff S. SIFT: Predicting amino acid changes that affect protein function. Nucleic Acids Res. 2003 Jul 1;31(13):3812–4.
5. Adzhubei I, Jordan DM, Sunyaev SR. Predicting functional effect of human missense mutations using PolyPhen-2. Curr Protoc Hum Genet. 2013 Jan;Chapter 7:Unit7.20.
6. González-Pérez A, López-Bigas N. Improving the assessment of the outcome of nonsynonymous SNVs with a consensus deleteriousness score, Condel. Am J Hum Genet. 2011 Apr 8;88(4):440–9.
7. Rentzsch P, Witten D, Cooper GM, Shendure J, Kircher M. CADD: predicting the deleteriousness of variants throughout the human genome. Nucleic Acids Res. 2019 Jan 8;47(D1):D886–94.
8. Ioannidis NM, Rothstein JH, Pejaver V, Middha S, McDonnell SK, Baheti S, et al. REVEL: An Ensemble Method for Predicting the Pathogenicity of Rare Missense Variants. Am J Hum Genet. 2016 Oct 6;99(4):877–85.
9. Dong C, Wei P, Jian X, Gibbs R, Boerwinkle E, Wang K, et al. Comparison and integration of deleteriousness prediction methods for nonsynonymous SNVs in whole exome sequencing studies. Hum Mol Genet. 2015 Apr 15;24(8):2125–37.
10. Reva B, Antipin Y, Sander C. Predicting the functional impact of protein mutations: application to cancer genomics. Nucleic Acids Res. 2011 Sep 1;39(17):e118.
11. Takeda JI, Nanatsue K, Yamagishi R, Ito M, Haga N, Hirata H, et al. InMeRF: prediction of pathogenicity of missense variants by individual modeling for each amino acid substitution. NAR Genom Bioinform. 2020 Jun;2(2):lqaa038.
12. Samocha KE, Kosmicki JA, Karczewski KJ, O'Donnell-Luria AH, Pierce-Hoffman E, MacArthur DG, et al. Regional missense constraint improves variant deleteriousness prediction [Internet]. 2017 [cited 2024 May 2]. Available from: http://biorxiv.org/lookup/doi/10.1101/148353
